# RUNX1-MUC13 Interaction Activates Wnt/β-Catenin Signaling Implications for Colorectal Cancer Metastasis

**DOI:** 10.7150/ijbs.98396

**Published:** 2024-09-16

**Authors:** Xinyi Chen, Jingyao Tu, Mu Yang, Yuan Wang, Bo Liu, Hong Qiu, Xianglin Yuan

**Affiliations:** Department of Oncology, Tongji Hospital, Tongji Medical College, Huazhong University of Science and Technology, Wuhan, Hubei, China.

**Keywords:** Colorectal Cancer, Liver Metastasis, MUC13, RUNX1, Transcriptional Regulation

## Abstract

**Background:** Colorectal cancer (CRC) remains a significant global health challenge, often characterized by late-stage metastasis and poor prognosis. The Runt-related transcription factor 1 (RUNX1) plays a dual role as both an oncogene and a tumor suppressor in various cancers, including CRC. However, the specific regulatory mechanisms of RUNX1 in CRC, particularly its direct roles, are not fully understood.

**Objective:** This study aimed to investigate the role of RUNX1 in CRC progression and its interaction with Mucin 13 (MUC13) as a potential regulatory target.

**Methods:** RUNX1 expression was analyzed in CRC tissues and cell lines compared to controls. *In vitro* and *in vivo* assays were conducted to assess the effects of RUNX1 overexpression and knockdown on cell behavior. ChIP-seq and RNA-seq analyses were performed to identify RUNX1 targets, with a focus on MUC13.

**Results:** RUNX1 expression was significantly upregulated in CRC tissues and cells, correlating with advanced pathological characteristics and poor patient outcomes. RUNX1 overexpression enhanced CRC cell proliferation, migration, invasion, and G2/M phase arrest, while its knockdown had the opposite effects. MUC13 was identified as a direct transcriptional target of RUNX1, with its expression contributing to the activation of the Wnt/β-catenin signaling pathway. Disruption of MUC13 partially reversed the malignant phenotypes induced by RUNX1.

**Conclusion:** RUNX1 promotes CRC progression by upregulating MUC13 and activating the Wnt/β-catenin pathway. This RUNX1-MUC13 axis represents a potential therapeutic target for managing CRC.

## Introduction

According to the most current data, colorectal cancer (CRC) ranks as the third most diagnosed cancer globally and the second leading cause of cancer-related death 1. Current clinical strategies encompass surgery, chemotherapy, and radiotherapy, yet the prognosis for CRC patients, particularly those with metastatic CRC (mCRC), remains unsatisfactory^2^. In mCRC, liver metastases are the most common, occurring in 50-60% of cases, followed by lymph nodes (35-40%), lungs (10-30%), and the peritoneum (5-20%) 3. Current data suggest that upwards of 25% of patients initially present with colorectal liver metastasis (CRLM), with liver metastasis developing in approximately half of all colorectal cancer patients eventually 4. Therefore, understanding the molecular underpinnings of colorectal cancer metastasis is vital for advancing more effective therapeutic approaches.

Runt-related transcription factor 1 (RUNX1), also known as acute myeloid leukaemia 1, is a constituent of the RUNX family of transcription factors (RUNX1, RUNX2 and RUNX3), which is characterized by its evolutionary conservation and critical role as lineage determinants across diverse tissues 5,6. While RUNX1 is well-recognized for its impact on hematological malignancies 7, recent research has illuminated its paradoxical role in solid tumors, including its ambiguous function in colorectal cancer.

Aberrant over-expression of RUNX1 has been noted in various cancer types, suggesting both oncogenic and tumor-suppressive roles. In epithelial ovarian carcinoma 8, clear cell renal cell carcinoma 9, glioma 10, gastric cancer 11, and pancreatic cancer 12, RUNX1 has been linked to tumor progression. An initial microarray study differentiating between adenocarcinoma metastases and primary tumors identified Runx1 as part of a 17-gene signature associated with metastasis 13. Fritz *et al.* have demonstrated the significance of the RUNX family transcription factors in the regulation of epithelial-mesenchymal transition (EMT) and tumor stemness pathways in breast cancer 14, both of which are closely linked to the development of a highly aggressive tumor phenotype. Similarly, enhanced lung metastasis due to RUNX1 overexpression in an endometrial murine model indicates RUNX1's critical influence on distant metastasis in malignancies 15. Research by Wang *et al.* revealed enhanced expression of RUNX1 in tissues of hepatocellular carcinoma, promoting proliferation, invasion, and metastasis of HCC cells through the COL4A1/FAK/Src signaling cascade 16. Conversely, there is a loss of RUNX1 expression in the development of luminal estrogen-receptor-positive (ER+) breast cancer, pointing to a tumor-suppressive function 17. Further investigations have solidified RUNX1's predominantly tumor-suppressive capacity in ER+ breast cancer by counterbalancing the estrogen-driven suppression of AXIN1, an integral component of the Wnt/β-catenin signaling pathway 18. Bridges *et al.* elucidated that RUNX1 deletion increases the incidence of ovarian tumors in mice by impairing granulosa cell differentiation and modulating the expression of critical genes within the Wnt/β-catenin pathway 19. Notably, several studies have explored the role of RUNX1 in CRC. It is particularly concerning that RUNX1 is capable of activating the TGF-β signaling pathway. Given the critical role of the TGF-β signaling pathway in the EMT process in CRC, its enhancement can facilitate migration and invasion of CRC cells 20. Conversely, RUNX1's tumor-suppressive role is notably crucial within the gastrointestinal tract, effectively reducing the incidence of tumorigenesis 21. RUNX1 manifests dichotomous roles, reflecting the complexity of its regulatory mechanisms in cancer development. However, the precise role and mechanisms of RUNX1 in CRC remain elusive, prompting the comprehensive explorations detailed in this study.

In a preceding study, the F3-Caco-2 cell line (referred to as F3, following three rounds of *in vivo* selection of Caco-2) was successfully developed, displaying pronounced migration, invasion, and liver metastasis capabilities 22. High-throughput RNA sequencing was performed on three pairs of the parent Caco-2 cell line (henceforth referred to as F0) and the evolved F3 cell line to explore potential targets influencing CRC progression. This study uniquely identified Mucin 13 (MUC13) not only as a significantly upregulated gene but also elucidated its regulation by RUNX1—a novel finding in the context of CRC. MUC13 emerged as one of the most significantly upregulated genes in the F3 cell line, as demonstrated by the high-throughput RNA-seq analysis 22. MUC13, a glycoprotein from the mucin family, is essential for protecting, lubricating, and hydrating the apical surfaces of epithelial cells in the gastrointestinal and respiratory tracts 23. Additionally, MUC13 expression is observed to elevate in the context of infection and inflammation 24. Aberrant expression of MUC13 has been observed in various solid tumors, including hepatocellular carcinoma 25, gastric cancer 26, pancreatic cancer 27, renal cancer 28, ovarian cancer 29, and lung cancer 30, suggesting that MUC13 may have a cancer-promoting effect. Particularly noteworthy is that previous research has shown that MUC13, through its interaction with GCNT3, activates the GSK3β/β-catenin signaling pathway, promoting hepatocellular carcinoma progression 31, a key focus of this study. Additionally, it has been demonstrated that the overexpression of MUC13 significantly correlates with poor prognosis in hepatocellular carcinoma, attributed to its role in activating Wnt signaling 25. However, the mechanisms driving colorectal cancer metastasis through MUC13 have not been well elucidated, and the interplay between MUC13 and RUNX1, both critical in CRC and liver metastasis, has not been established.

In this study, we conducted a comprehensive analysis of RUNX1 expression in colorectal cancer tissues, compared to adjacent normal tissues, using both clinical samples and online databases. Our findings indicated a notable increase in RUNX1 levels in cancerous tissues, associated with more aggressive tumor characteristics and a poorer prognosis. Comprehensive *in vitro* and *in vivo* studies revealed that RUNX1 substantially enhances cell proliferation, invasion, and migration, thus contributing critically to the progression and metastasis of colorectal cancer. Crucially, integrative ChIP-Seq and RNA-Seq analyses identified MUC13 as a direct transcriptional target of RUNX1, a novel insight that refines the understanding of CRC pathogenesis. The binding of RUNX1 to the MUC13 promoter was demonstrated to initiate the Wnt/β-catenin signaling pathway, thereby facilitating EMT. This interaction correlates with worsened disease outcomes and higher mortality rates, underscoring the potential of the RUNX1/MUC13 pathway as a target for therapeutic intervention in colorectal cancer. Significantly, a positive correlation was observed between the protein expression levels of RUNX1 and MUC13 in CRC tumor tissues. Summarily, this study thoroughly investigates the mechanisms and clinical relevance of RUNX1 and MUC13 in CRC, highlighting these molecules as promising targets for therapeutic development.

## Materials and Methods

### Tissue microarray and immunohistochemistry

The study utilized a colorectal cancer tissue microarray (TMA, HColA180Su13), sourced from Shanghai Outdo Biotech Co., Ltd., China. It contained 90 colon adenocarcinoma tissues accompanied by 90 corresponding adjacent normal colon samples. The TMA slides underwent a dewaxing process, followed by rehydration. Subsequently, the procedure involved incubating with 0.3% H_2_O_2_ for 30 minutes to deactivate endogenous peroxidase. Subsequent to antigen retrieval and serum blocking, the primary antibody was incubated overnight at 4°C. The slides were rinsed and then incubated with a secondary antibody labeled with horseradish peroxidase for an hour. Finally, utilizing 3,3'-diaminobenzidine (DAB) as the chromogenic agent, the samples were microscopically assessed. Following immunohistochemistry (IHC), TMA slides were scanned using Aperio ScanScope (Aperio Technologies, Vista, CA) to acquire digital images, subsequently analyzed with Aperio Image Scope software (Aperio Technologies, Vista, CA). Ethical guidelines were strictly followed, with all participants providing informed consent, and the study was sanctioned by the ethics committee of Shanghai Outdo Biotech Co., Ltd.

### IHC score assessment

The evaluation of immunostaining for each TMA core was conducted independently by two clinical pathologists, blind to the pathological and clinical background, using a light microscope and a standardized classification system. Based on the percentage of positive tumor cells, the staining extent was graded as 0 (0%), 1 (1-25%), 2 (26-50%), 3 (51-75%), or 4 (>75%). The intensity of staining was scored as 0 (no staining), 1 (weak staining), 2 (moderate staining), and 3 (strong staining). The final RUNX1 and MUC13 immunohistochemical score was determined using the following formula: overall score = extent score × intensity score.

### Patients

Pathological sections from fifteen individuals with CRLM were retrieved from Tongji Hospital's Department of Pathology in China. The tumor areas were subjected to immunohistochemical analysis using anti-RUNX1 (ab23980; Abcam) and anti-MUC13 (ab235450; Abcam) antibodies. All conducted experiments received approval from the Clinical Ethics Committee at Huazhong University of Science and Technology.

### Online data collection

Utilizing the TCGA platform (https://portal.gdc.com), RNA-sequencing expression profiles for CRC, along with related clinical data, were sourced. Post accessing the Gene Expression Omnibus (GEO) database (http://www.ncbi.nlm.nih.gov/geo/), bioinformatics analysis incorporated five CRC datasets, specifically GSE14297, GSE37182, GSE44861, GSE71187, and GSE49355, focusing on comparing RUNX1 and MUC13 levels in cancerous versus non-cancerous tissues. TIMER (https://cistrome.shinyapps.io/timer/) and GEPIA (http://gepia.cancer-pku.cn/) online tools were employed to detect the expression levels of target genes in pan-cancer. Investigating the protein expression levels of RUNX1 and MUC13 in CRC involved utilizing IHC data from the Human Protein Atlas (HPA) database (https://www.proteinatlas.org/) and mass spectrometric data from the Clinical Proteomic Tumor Analysis Consortium (CPTAC) database (https://cptac-data-portal.georgetown.edu/cptacPublic/). A survival analysis was performed using the GEPIA database, followed by the generation of Kaplan-Meier curves.

### Cell culture and reagents

Human CRC cell lines (HCT 116, HT-29, SW480, Caco-2, and LoVo), normal human colon epithelial cell line (NCM460), human HCC cell lines (Huh7, HepG2, Hep3B, SK-Hep1, and MHCC97-H), along with normal liver cell lines (LO-2), procured from the American Type Culture Collection (ATCC; Manassas, VA), were cultured in humidified conditions at 37 °C with a 5% CO2 atmosphere. Growth medium used was DMEM (Gibco), supplemented with 10% FBS (Gibco) and 1% Pen/Strep solution.

XAV-939 (catalog no. HY-15147) and CHIR-99021 (catalog no. HY-10182), sourced from MedChemExpress (Shanghai, China), were utilized to investigate β-catenin dynamics within the Wnt signaling pathway. XAV-939 inhibited β-catenin accumulation; CHIR-99021 acted as a GSK-3 inhibitor to stabilize β-catenin.

### *In vivo* metastatic model and bioluminescence imaging

Six-week-old male BALB/c nude mice, sourced from GemPharmatech Co., Ltd (Jiangsu, China), with an initial maternal body weight ranging from 18 to 20 grams across all groups, were maintained in accordance with the National Institutes of Health guidelines for animal care. Approval for this investigation was provided by the Tongji Hospital Institutional Review Board (Ethic Approval Number: TJH-202001006). The mice, initially weighing between 18 and 20 grams, were randomly assigned to different experimental groups to ensure unbiased results. For inducing liver metastases, intrasplenic injections were administered to the mice. Each mouse was anesthetized with 1.5% pentobarbital sodium at 50mg/kg dosage, followed by a small abdominal incision to access the spleen. Luciferase-expressing lentivirus-transduced Caco-2 cells, in a 1×10^^6^ cell suspension of 100 μL PBS with luciferase-labeled Caco-2 cells (Caco2—LUC), were injected into six-week-old male athymic nude mice spleens. To control hemorrhaging and minimize the risk of tumor cell dissemination, gentle pressure was applied to the puncture site for a duration of one minute using a cotton swab. After carefully reinserting the spleen into the abdominal cavity, 4-0 sterile, nonabsorbable surgical suture thread was used to suture the incision. Following 30 days post-induction, observation of tumor presence occurred, through detection of luciferase activity in the live animal with the Lago X *in vivo* imaging system (Spectral Instruments Imaging). Administration of anesthesia involved intraperitoneal injection of pentobarbital sodium (1.5%, 50mg/kg), then the intraperitoneal administering of 150 mg/kg D-luciferin. Ten minutes following injection, images were obtained using the Lago X. Each photograph was captured within a duration of 30 seconds. Following the euthanasia of the mice, liver specimens were harvested and then fixed in newly prepared 4% paraformaldehyde. The ImageJ software facilitated calculation of liver pixel area and metastatic lesion pixel area, enabling determination of the metastasis area percentage. Animal care was conducted in a pathogen-free environment and in accordance with our institution's guidelines.

### Mice xenograft models

Male BALB/c nude mice aged 4-6 weeks were procured from GemPharmatech Co., Ltd (Jiangsu, China). 5 × 10^6^ cells stably expressing either a control vector, RUNX1(MUC13)-OE, or RUNX1(MUC13)-shRNA, were resuspended in 200 μL of PBS for subcutaneous administration into the right flanks of mice. At five-day intervals, tumor volumes were gauged with calipers and computed with the equation: Volume (mm^3^) = length × width^2^/2. One month post-injection, euthanasia was administered to all mice. Tumors were excised, weighed, photographed, and then preserved in 4% paraformaldehyde for subsequent analysis.

### Lentiviral vectors and transfection

Lentiviral vectors encompassing RUNX1 shRNA, RUNX1 overexpression, MUC13 shRNA, and MUC13 overexpression were procured from Obiosh Biotechnology (Shanghai, China). The specific sequences of the target short hairpin RNA (shRNA) are displayed in Supplementary Table 1. Procedures for lentiviral infection adhered to the guidelines provided by the manufacturer.

### Cell cycle analysis

Analysis of the cell cycle was conducted on the transfected cells. These cells underwent fixation in ice-cold 70% ethanol for at least 2 hours at 4 °C. Subsequently, the samples were stained in the dark for 30 min at 37 °C with 100 μL propidium iodide (100 μg/mL PI; Beyotime, China). Finally, the cells were analyzed using the CytoFlex (Beckman CytoFlex, USA). Further analyses of the cell cycle were conducted using ModFit LT3.2 software.

### Evaluation of cell apoptosis

Cell apoptosis was evaluated utilizing the FITC Annexin V/PI Apoptosis Detection Kit (BD Pharmingen, USA) as per the specified manufacturer's protocol. Initially, cells were collected, rinsed with Binding Buffer at 4˚C, and subsequently resuspended in 200 μL of the same buffer. A sample of 1 × 10^^5^ cells was then labeled with 5 μL of both Annexin V-FITC and PI and incubated in the staining solution at room temperature away from light. Analysis involved processing 10,000-20,000 cells through the CytoFLEX flow cytometer (Beckman Coulter, Brea, CA, USA) and employing FlowJo V10.8.1 software for data interpretation.

### Cell wound healing assay

Approximately 1 × 10^6^ cells were inoculated into each well of six-well culture plates and incubated for 24 h, reaching a confluence level of 80-90%. Using a 10 μl plastic pipette tip, scratch wounds were generated, followed by the cultivation of cells in DMEM containing 2% FBS. Photographs were taken of the wound margins, and the process of migration was observed and tracked 48 h after wound formation. To quantify cell motility, the advancing edges of cells were measured for the distance in three randomly picked microscopic fields (×200) during each time interval.

### Cell counting kit-8 (CCK-8) assay

Following the manufacturer's protocol, the impact of RUNX1 and MUC13 on cell proliferation was evaluated using a CCK-8 assay (HY-K0301, MCE, China). A density of 3 × 10^^3^ cells per well was seeded into 96-well plates, each well containing 200 μL of culture medium. The supernatant was removed, and 10 μL of CCK-8 reagent together with 100 μL of medium was added to each well at 24 h, 48 h, 72 h, and 96 h intervals. Absorbance at 450 nm was measured after a 2-hour incubation at 37 °C in the dark using a microplate reader (BioTek ELx800, USA). All experiments were performed in triplicate.

### Transwell assay

In the Transwell assay, chambers with an 8 μm pore size (#3422, Corning, Costar, NY, USA) were utilized to assess cell migration and invasion. The upper chamber was pre-coated with Matrigel (BD Biosciences, USA) to serve as a matrix barrier. In the upper chamber, 2 × 10^^4^ cells per well were seeded in 200 µL of FBS-free DMEM, while the lower chamber was filled with 500 µL of DMEM containing 10% FBS. After an 18-hour incubation, cells present in the lower chamber were fixed with 4% paraformaldehyde, and cells remaining on the upper membrane were removed. Migrated cells on the underside of the membrane were stained with 1% crystal violet and counted (five random microscopic fields per well) using an inverted microscope (SDPTOP, China).

### EdU proliferation assay

Cell proliferation was evaluated using the Cell-Light^TM^ Apollo 488 Stain Kit (C10371-3, RiboBio Corporate, Guangzhou, China). After transfection, cells were seeded into 96-well plates at a density of 5 × 10^^3^ cells per well and allowed to attach overnight. The following day, 50 μM EdU labeling medium was added, and the cells were incubated for 2 hours. Cells were then fixed with 4% paraformaldehyde (PFA), neutralized with 2 mg/mL glycine, and permeabilized with 0.5% Triton X-100 for 30 minutes. After washing with PBS, the cells were stained with 1X Apollo reaction buffer and Hoechst33342, and fluorescence micrographs were captured using a Leica DMI3000B inverted microscope.

### Western blot analysis

Total proteins were extracted using RIPA buffer, which was enhanced with 1% PMSF. Protein concentrations were determined through the use of a BCA protein assay kit (BOSTER, Wuhan, China), in accordance with the manufacturer's instructions. Proteins were separated by electrophoresis at 70 V in the stacking gel and 100 V in the resolving gel for 120 minutes, and then transferred to PVDF membranes at 200 mA for 120 minutes with 20% methanol. Membranes were blocked with 5% non-fat skim milk in TBS-T buffer and incubated overnight at 4°C with primary antibodies against RUNX1 (Abcam, ab23980), MUC13 (Abcam, ab235450), E-Cadherin (CST, #14472), Vimentin (Proteintech, 10366-1-AP), N-Cadherin (CST, #13116), β-Catenin (CST, #8480), GSK-3β (CST, #12456), Phospho-GSK-3β (CST, #5558), and GAPDH (Proteintech, 60004-1-Ig). After primary antibody incubation, membranes were washed with TBST and incubated with secondary antibodies (HRP-goat anti-rabbit IgG or goat anti-mouse IgG, diluted 1:10000) for 1 hour at room temperature. Proteins were visualized using an enhanced chemiluminescence (ECL) kit (Thermo Fisher Scientific) and detected with a G: BOX Chemi X system (Syngene).

### Quantitative real-time PCR

Total RNA was extracted from tissues and cell cultures using TRIzol reagent (Takara, Japan), and its concentration was determined with a NanoDrop 2000 instrument (ThermoFisher Scientific). Subsequently, cDNA synthesis was carried out using a S1000 Thermal Cycler (Bio-Rad, USA). Quantitative PCR employed a 2× SYBR Green qPCR Master Mix (low ROX) (Bimake, China) in a 7900HT Fast Real-Time PCR System (ThermoFisher Scientific), with primers supplied by TSINGKE (Beijing, China). The thermocycler conditions were as follows: Hot-Start DNA Polymerase Activation at 95.0°C for 30 sec, followed by 40 cycles of denaturation at 95.0°C for 15 sec, annealing at 60.0°C for 30 sec, and extension at 72.0°C for 30 sec. Melt curve analysis was performed with one cycle of 95.0°C for 15 sec, 60.0°C for 60 sec, and 95.0°C for 15 sec. The qRT-PCR data were analyzed using the 2^-ΔΔCT^ calculation method.

### ChIP-seq data analysis

ChIP-Seq experiments, executed by SEQHEALTH Biotech (Wuhan, China), consisted of cell cross-linking with formaldehyde, lysis for nuclear material release, and immunoprecipitation using the RUNX1 primary antibody from Abcam (ab23980) to isolate protein-DNA complexes. This was followed by DNA fragmentation (200-1000 bp) and purification, culminating in the construction of sequencing libraries as per the BGISEQ-500 ChIP Seq protocol. Library preparation involved end-repair, A-tailing, adaptor ligation, and PCR amplification. Quality checks were conducted using agarose gel electrophoresis and Qubit 2.0. Post deep sequencing, data alignment with the human genome (hg19) enabled analysis of RUNX1-associated protein-DNA interactions, including whole-genome peak calling and motif analysis. Western blotting was utilized for verifying protein presence, providing a comprehensive insight into the binding characteristics of RUNX1.

### Co-immunoprecipitation (Co‑IP) assay

In the Co-IP assay, cells were rinsed with PBS and lysed in a buffer composed of NP-40 and PMSF at a 100:1 ratio, with 100-200 μL used per sample. Following a 30-minute lysis on ice and centrifugation at 12,000 rpm for 20 minutes at 4°C, a portion of the supernatant was mixed with 5× protein loading buffer at a 4:1 ratio, boiled for 5 minutes, and designated as 'Input'. The remaining the supernatant was divided, each aliquot receiving 1 μg of IgG and 50 μL Protein A/G Magnetic Beads, and incubated at 4°C for three hours to reduce non-specific binding. After magnetic separation, lysates were transferred to new tubes; one tube was treated with 2-4 μg of target protein antibodies (anti-RUNX1 and anti-MUC13), and another tube with IgG as a control. Following an overnight incubation at 4°C, 50 μL Protein A/G Magnetic Beads were added for two hours to capture protein-antibody complexes. After separation, the supernatant was discarded, and the bead-antibody-protein complexes were washed five times with lysis buffer and resuspended in 20 μL of 1× protein loading buffer, boiled for 5 minutes to denature, and prepared for Western Blot analysis.

### Dual-luciferase assay

To ascertain the direct regulatory interaction between RUNX1 and MUC13, the promoter region of MUC13 harboring either the hypothesized RUNX1 binding site or a modified variant was cloned into a luciferase reporter construct, such as pmiR-RB-REPORT™. Approximately 1 × 10^^5^ cells per well were seeded in 24-well plates. Post 24 hours, cells were co-transfected with reporter plasmids combined with either RUNX1 expression vectors or the corresponding control vectors using Lipofectamine 3000 (Invitrogen). After 48 hours of incubation, cellular lysates were prepared using Passive Lysis Buffer (Promega), and luciferase assays were performed with the Dual-Glo^®^ Luciferase Assay System (Promega) on a luminometer. Firefly luciferase readings were adjusted relative to Renilla luciferase, and the data were displayed as the ratio of Firefly to Renilla luciferase activities.

### H&E staining

Fixation of liver specimens from nude mice utilized 4% paraformaldehyde. Sections, 5 μm thick and paraffin-embedded, were then dewaxed in xylene I and II. Post-deparaffinization, a graded series of ethanol (100%, 95%, 85%, and 75%) immersion was performed on each section for 5 minutes, followed by water rinse. Subsequently, sections were bathed in distilled water. Subsequent to hematoxylin-eosin staining, sections underwent sequential immersion in 95% ethanol, followed by two absolute ethanol stages for 5 minutes each, clearing in xylene I and II, and drying. Neutral gum was applied for sealing the sections. Microscopic examination was employed to detect hepatic metastatic lesions.

### Immunohistochemistry

Immunohistochemistry was performed to determine RUNX1, MUC13, and Ki67 expression. Tissue sections underwent xylene and graded ethanol deparaffinization, rehydration, and endogenous peroxidase blocking with 3% H2O2. Antigen retrieval was achieved in citrate buffer via microwave heating. After blocking with goat serum, sections were incubated overnight at 4°C with primary antibodies against RUNX1 (ab23980, Abcam), MUC13 (ab235450, Abcam), and Ki67 (Hubei Bioassay Biotechnology Co., Ltd.). HRP-conjugated secondary antibodies (Wuhan Qi Dong Promoter Biological Co., Ltd.) were subsequently applied, followed by development with diaminobenzidine. Hematoxylin was used for counterstaining, and slides were examined under a light microscope.

### Immunofluorescence examinations

EMT markers were identified in F0 and F3 cells and in liver metastases from mice, through immunofluorescence assays.

#### Cell Immunofluorescence

Upon reaching approximately 70% confluence in 24-well plates, cells underwent washes with PBS, fixation in 4% paraformaldehyde, and permeabilization with 0.5% Triton X-100. Following blocking with 5% BSA, cells were incubated overnight at 4°C with primary antibodies, specifically E-cadherin (14472, CST) and vimentin (10366-1-AP; Proteintech), followed by treatment with fluorescent secondary antibodies. Nuclei were highlighted with DAPI staining for fluorescence microscopy imaging.

#### Tissue Immunofluorescence

Paraffin-embedded liver metastasis samples were initially deparaffinized, then rehydrated sequentially using a series of ethanol solutions, each for five minutes, and finally rinsed in double distilled water. Antigen retrieval was performed using citrate buffer at pH 6.0, autoclaved for two minutes, then allowed to cool to ambient temperature. Subsequently, the tissues underwent a triple wash with TBS, followed by a 20-minute room temperature incubation in 10% normal donkey serum as a blocking agent. The sections underwent overnight incubation at 4 °C with primary antibodies E-cadherin (14472, CST) and vimentin (10366-1-AP; Proteintech) at a 1:400 dilution in 2% BSA in PBST, followed by a 30-minute incubation at 37°C with Alexa Fluor®594-conjugated secondary antibodies (donkey anti-rabbit, A21206, Life Technologies), shielded from light. Sections were mounted using Prolong Gold Antifade Reagent containing DAPI, and images were acquired with K-Viewer software.

### Statistical analysis

The unpaired two-tailed Student's t-test was used for data analysis. Multiple testing involved determining *P* values through a two-way analysis of variance with Bonferroni post-tests. Disparities in RUNX1/MUC13 expression between tumor and adjacent colon tissues were evaluated using the Wilcoxon rank-sum test and Kruskal-Wallis test for tripartite comparisons. The association between RUNX1/MUC13 expression status and clinical-pathological features was examined using the Chi-square test. Survival analysis was conducted using Kaplan-Meier and log-rank tests. Two-way ANOVA, incorporating Bonferroni post-tests for multiple comparisons, assessed RUNX1 IHC scores in liver metastasis and surrounding tissues. RNAseq data from TCGA were processed using R software (v4.0.3), GSVA, and Spearman correlation to analyze associations between RUNX1 and EMT markers. Statistical analyses were conducted using GraphPad Prism 9.0 and IBM SPSS Statistics (Version 27). Continuous variables were expressed as mean ± SEM. *P* value < 0.05 was considered statistically significant.

## Results

### RUNX1 is overexpressed and indicates poor prognosis in colon cancer

**Figure 1:**
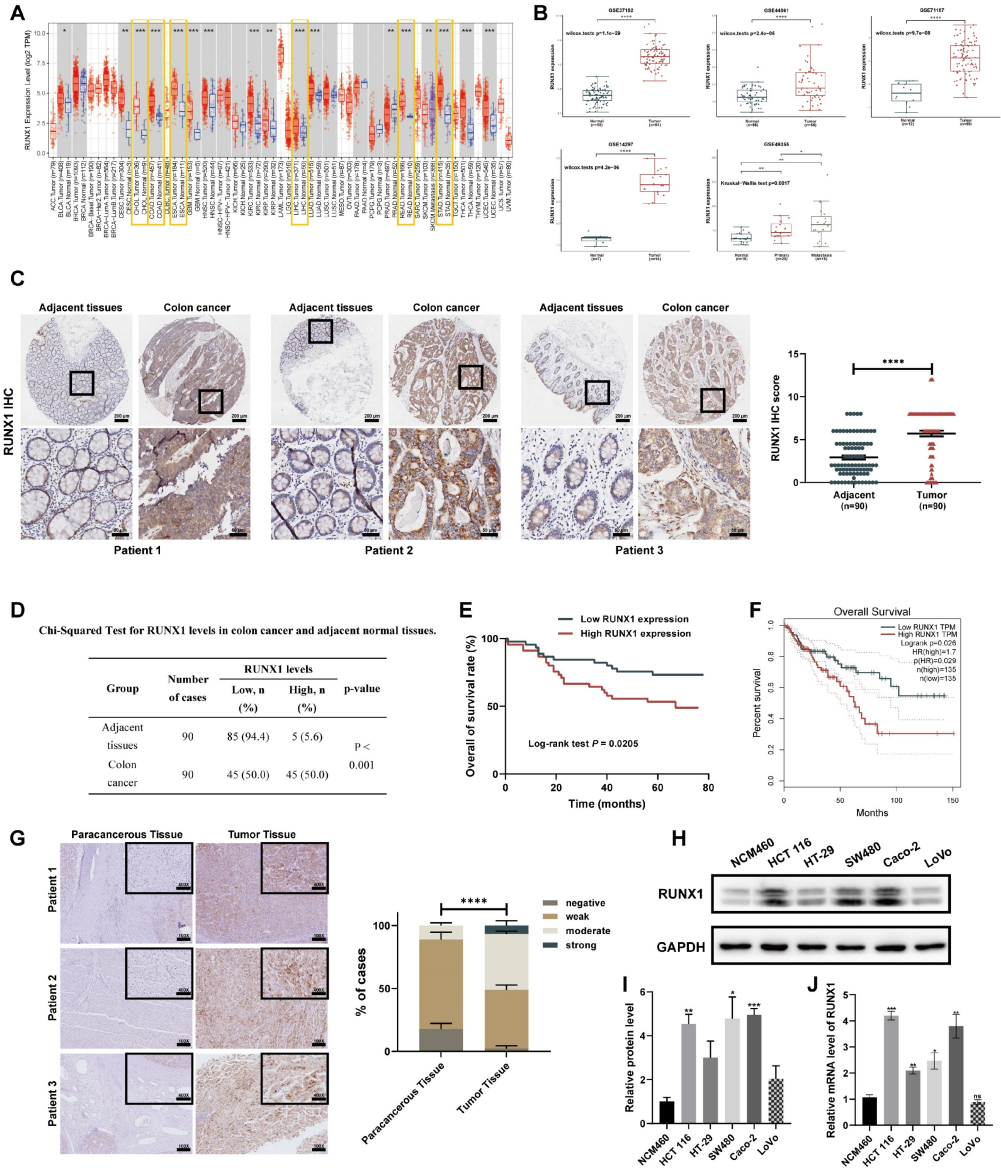
** RUNX1 is upregulated in CRC samples and cells. (A)** Based on the TIMER database, differences in the expression levels of RUNX1 in diverse digestive system malignancies. **(B)** Validation in GEO datasets (GSE37182, GSE44861, GSE71187, GSE14297, GSE49355) confirmed RUNX1 mRNA upregulation in CRC. **(C)** A TMA, encompassing 90 CRC tumor and corresponding adjacent colon tissues, was subjected to RUNX1 IHC staining (left). Analysis of RUNX1 staining levels between CRC and adjacent tissues was statistically conducted (right). Scale bar: 200 μm and 50 μm, respectively. **(D)** A chi-squared test determined the expression disparity of RUNX1 in CRC compared to normal tissues. **(E)** Kaplan-Meier method and log-rank test were used to analyze the correlation between RUNX1 expression levels and the OS of CRC patients. The median value of RUNX1 expression served as the threshold for low/high classification. **(F)** Kaplan-Meier analysis, utilizing GEPIA data, assessed COAD patients' survival relative to RUNX1 expression. **(G)** Immunohistochemical detection highlighted RUNX1 protein expressions in clinical CRLM tumor and the adjacent non-cancerous tissues. Scale bars: 100X = 200 μm; 400X = 100 μm. Quantitation on the right: Pathological evaluation of RUNX1 expression between CRLM tumor tissues and adjacent noncancerous tissues. Data underwent two-way ANOVA processing, indicating high significance: ***P < 0.001. **(H)** Western blot analysis displaying RUNX1 expression across five CRC cell lines and the NCM460 normal human colonic epithelial cell line. **(I)** Quantitative evaluation of RUNX1 protein levels derived from Western blot data. **(J)** qPCR determination of RUNX1 mRNA levels in the same cell lines as shown in (H). Error bars indicate mean ± SEM, derived from three independent experiments. Survival curves underwent assessment via the log-rank test. ns, not significant; *P < 0.05; **P < 0.01; ***P < 0.001; ****P < 0.0001.

To establish the broader oncogenic potential of RUNX1 across various cancers, we analyzed its expression patterns utilizing TCGA data through computational platforms TIMER and GEPIA. The results revealed that RUNX1 expression is substantially higher in the majority of digestive system tumors **(Figure 1A; Supplementary Figure 1A)**, suggesting a potential pro-oncogenic function of RUNX1 in the human digestive system. To further verify the expression level of RUNX1 in CRC malignant tissues as compared to noncancerous tissues, we acquired five microarray datasets (GSE37182, GSE44861, GSE71187, GSE14297, and GSE49355) from the GEO datasets. As a result, when comparing the CRC samples to normal samples, it was observed that the RUNX1 expression levels were substantially upregulated in the five GEO datasets **(Figure 1B)**. Besides, the expression level of RUNX1 further increased in colon tumor with liver metastasis compared with primary colon tumor in GSE49355 dataset **(Figure 1B)**, indicating that the higher malignant tumors were associated with the higher RUNX1 expression levels. To investigate the involvement of RUNX1 in CRC, we analyzed its protein expression levels on a tissue microarray (TMA) comprised of 90 cases and their matched adjacent normal tissues. This was accomplished by evaluating the expression of RUNX1 through IHC staining scoring. Consistent with these biostatistics and the previous study 32, RUNX1 was significantly elevated in tumor tissues relative to levels in the corresponding adjacent non-tumor tissues **(Figures 1C, D)**. Protein expression data obtained from Clinical Proteomic Tumor Analysis Consortium (CPTAC) and Human Protein Atlas (HPA) databases confirmed the same conclusion **(Supplementary Figures 1B, C)**. Further, CRC patients were divided into two groups based on RUNX1 expression levels, followed by a Kaplan-Meier (KM) survival analysis. Notably, Kaplan-Meier survival analysis demonstrated that patients exhibiting high RUNX1 expression levels had poorer overall survival compared to patients with low levels of RUNX1 expression **(Figure 1E)**, consistent with the outcomes of the KM survival analysis using GEPIA database **(Figure 1F; Supplementary Figure 1D)**. Furthermore, we conducted an investigation into the association between RUNX1 expression and clinicopathological characteristics. The results revealed a significant correlation between higher RUNX1 expression and Grade (χ2 = 7.283, *P* = 0.007), AJCC Stage (χ2 = 11.072, *P* < 0.001), T stage (χ2 = 12.532, *P* < 0.001), N stage (χ2 = 8.598, *P* = 0.003) and Clinical outcome (χ2 = 5.657, *P* = 0.017)** (Table 1)**. Further assessment of RUNX1 protein expression was conducted through IHC staining on the pathological sections obtained from the Department of Pathology in Tongji Hospital.

The findings indicated that the majority of CRLM samples displayed higher protein expression of RUNX1 compared to corresponding paracancerous tissues, and quantification confirmed statistical significance **(Figure 1G)**.

To verify our findings above, we first detected the expression of RUNX1 in colon cancer cell lines (HCT 116, HT-29, SW480, Caco-2, and LoVo) and the normal human colonic epithelial NCM460 cells at mRNA and protein levels. Consistent with the preceding results, multiple CRC cell lines also expressed higher levels of the RUNX1 compared to the normal human colonic epithelial NCM460 cells in both mRNA and protein levels **(Figure 1H)**. Furthermore, since the liver is the most common metastatic site for CRC 33, and given the aforementioned findings indicating that hepatocellular carcinoma was also a type of digestive tumor characterized by abnormal upregulation of RUNX1 expression, we became intrigued about the alterations in RUNX1 expression observed in hepatocellular carcinoma cell lines. We detected the expression of RUNX1 in human normal liver cell line LO-2 and five available hepatocellular carcinoma (HCC) cell lines (Huh7, HepG2, Hep3B, SK-Hep1, and MHCC97-H), and results demonstrated a higher expression of RUNX1 in HCC cell lines, compared with LO-2, in both mRNA and protein levels **(Supplementary Figures 2A, B)**. Based on these findings, it can be concluded that the levels of RUNX1 protein are elevated in more aggressive CRC and predicts a poor prognosis of CRC, suggesting that RUNX1 may function as an oncogene in CRC development.

### RUNX1 promotes proliferation, migration, and invasion of CRC cells *in vitro*

**Figure 2:**
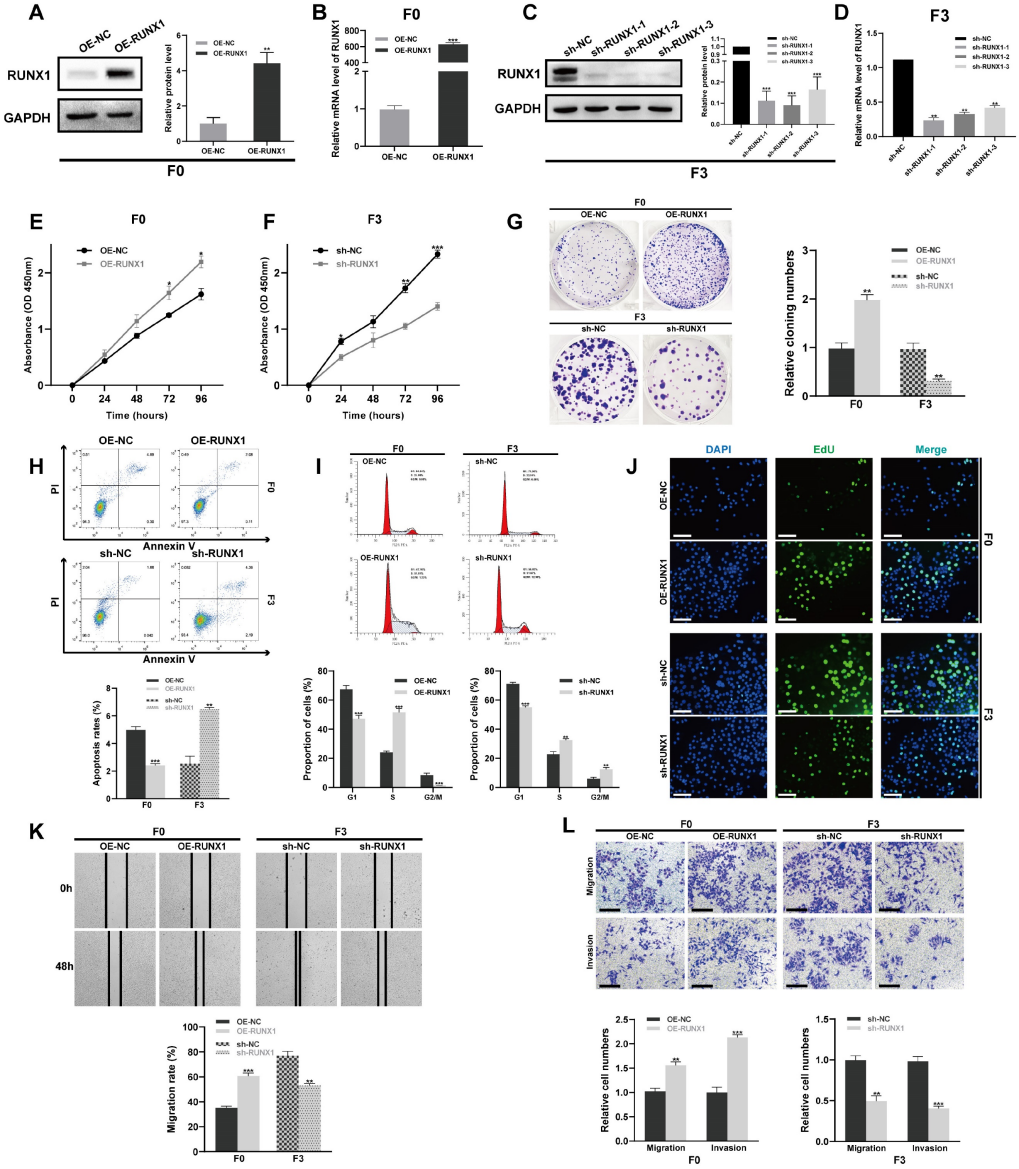
** RUNX1 promotes the invasive and migratory abilities of CRC cells *in vitro*. (A-D)** Overexpression and knockdown of RUNX1 in F0 and F3 cells were confirmed by qPCR and Western blot analyses. Quantitative analyses for protein levels are shown in bar graphs on the right of panels (A) and (C). **(E, F)** Cell viability subsequent to RUNX1 modulation was evaluated through the CCK-8 assay. **(G)** Colony formation was impacted by RUNX1 overexpression and knockdown. **(H)** Cell apoptosis post-RUNX1 modulation was evaluated by flow cytometry. **(I)** Alterations in cell cycle progression were observed following RUNX1 overexpression and knockdown. **(J)** Proliferative capabilities of cells after RUNX1 modulation were determined through EdU assay; scale bar: 100 µm.** (K, L)** RUNX1 manipulation's influence on cell migration and invasion was measured in F0 and F3 cells through wound healing and Transwell assays, respectively. Scale bars: 100 µm. The bar graphs (G, H, I, K and L) show the results of quantitative analyses. Error bars represent mean ± SEM of three independent experiments. *P < 0.05; **P < 0.01; ***P < 0.001.

Given the substantial upregulation of RUNX1 in CRC, which was associated with more advanced clinical stage and poorer overall survival, it is reasonable to postulate that such upregulation augments the aggressive behavior of CRC cells. To functionally characterize RUNX1's effects on CRLM, a CRC cell line (Caco-2 after three consecutive rounds of *in vivo* selection; hereafter referred to as F3) with enhanced migration and invasion capacities, along with pronounced liver metastatic potential, as previously confirmed 22, was employed in this research. Initially, we determined the levels of RUNX1 expression in both cell lines, to investigate whether RUNX1 has a significant contribution to CRC. In contrast, the highly invasive F3 exhibited a significant upregulation of RUNX1, whereas the poorly invasive parental Caco-2 (hereinafter referred to as F0) displayed a low-level expression of RUNX1 **(Supplementary Figures 3A, B)**. Investigation into RUNX1's biological impact on CRC progression entailed generating cell lines with stable ectopic overexpression and knockdown of RUNX1, through transfection of F0 and F3 cells with a RUNX1-overexpressing vector or a specific shRNA. qPCR and Western blotting analyses confirmed RUNX1 overexpression and knockdown, respectively **(Figures 2A-D)**. Subsequently, cell viability was determined in different groups with a CCK-8 assay. The results demonstrated that overexpression of RUNX1 increased F0 cells viability, conversely, knockdown of RUNX1 significantly decreased the viability of F3 cells **(Figures 2E, F)**. Furthermore, colony formation assay and flow cytometry demonstrated that overexpression of RUNX1 in F0 cells promoted cell proliferation while reducing cell apoptosis, whereas knockdown of RUNX1 resulted in opposing effects on F3 cells **(Figures 2G, H)**. Consistent with the observations described above, cell cycle distribution examination revealed that RUNX1 overexpression caused a shift in the cellular population from G2/M to S phase, whereas RUNX1 knockdown resulted in a notable increase of cells arrested in G2/M phase **(Figure 2I)**. Similarly, the findings from EdU assays revealed that RUNX1 overexpression markedly increased the number of EdU-positive cells, whereas RUNX1 knockdown yielded the converse effect ​**(Figure 2J)**. Next, wound healing, Transwell migration, and invasion assays were performed to investigate the impact of RUNX1 on CRC cell migration and invasion. Similarly, RUNX1 upregulation dramatically increased the migration and invasion capabilities of F0 cells, whereas RUNX1 inhibition resulted in a substantial reduction in the migration and invasion abilities of F3 cells **(Figures 2K, L)**. In conclusion, our results highlighted RUNX1's crucial role in promoting aggressive CRC phenotypes, indicating its potential relevance in CRC progression.

### RUNX1 facilitates the tumorigenesis and metastasis of CRC cells *in vivo*

**Figure 3:**
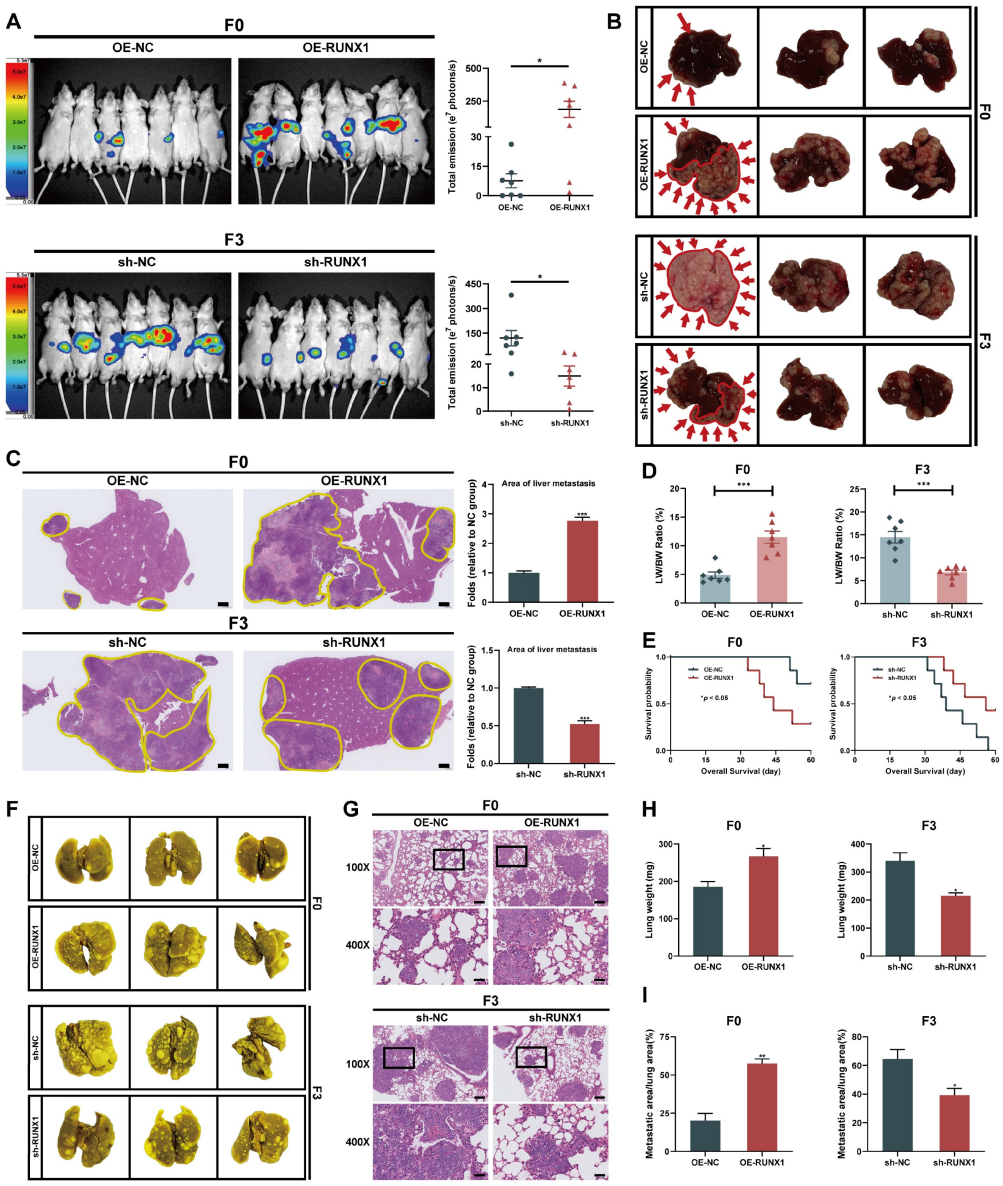
** RUNX1 facilitates metastasis of CRC cells to the liver and lung *in vivo*. (A)** Bioluminescence imaging conducted four weeks after intrasplenic injection illustrates liver metastasis, with quantified bioluminescent signals depicted on the right. **(B)** Representative macroscopic liver views with metastatic regions highlighted by red arrows and outlines to distinctly define areas of metastasis. **(C)** Representative sections depicting liver tissues and liver metastases stained with H&E, with corresponding quantitative data on the right. Metastatic regions are outlined by the yellow line. Scale bar: 500 µm. **(D)** Liver weight relative to body weight (LW/BW) ratio. **(E)** Kaplan-Meier survival analysis for the mice (Log-rank test, *P* < 0.05). **(F, G)** Representative images and corresponding H&E staining of lung tissue. Scale bars: 100X = 200 μm; 400X = 50 μm. **(H, I)** Quantification of the lung weight **(H)** and metastatic lung fraction **(I)** was conducted. Error bars represent mean ± SEM of three independent experiments. *P < 0.05; **P < 0.01; ***P < 0.001.

Considering RUNX1's enhancement of CRC cell migration and invasion *in vitro*, the study subsequently assessed RUNX1's impact on tumor growth and metastasis *in vivo*. First, either OE-RUNX1 (F0) or sh-RUNX1 (F3) cells (along with the respective negative control cells) were injected into the spleen of nude mice to construct liver metastasis model. Four weeks after intrasplenic injection, liver metastasis was monitored using bioluminescence imaging. The bioluminescence imaging revealed that ectopically overexpression of RUNX1 in F0 cells significantly promoted liver metastasis *in vivo*. In contrast, RUNX1 knockdown resulted in decreased metastatic spread of F3 cells to the liver **(Figure 3A)**. Consistent with the live *in vivo* imaging findings, macroscopic and histological observations also confirmed that RUNX1 upregulation increased area of metastatic lesions in liver **(Figures 3B, C)**. Furthermore, the ratio of liver weight to body weight (LW/BW) increased significantly in the groups with higher RUNX1 expression **(Figure 3D)**. Utilizing Kaplan-Meier analysis, observations indicated that augmented RUNX1 expression decreased mice's overall survival times, while its suppression improved their survival outcomes **(Figure 3E)**. To further elucidate the effect of RUNX1 on CRC metastasis *in vivo*, we established models of lung metastasis for CRC and subsequently quantified the lung metastatic lesions. Compared to the control group, RUNX1 overexpression resulted in a higher incidence of lung metastases, whereas RUNX1 knockdown significantly impeded the occurrence of lung metastases **(Figure 3F)**. The lung tissues of mice were dissected and subjected to H&E staining in order to identify the formation of lung metastases, the findings of which were in agreement with the macroscopic observations **(Figure 3G)**. Similarly, quantitative analysis revealed that mice in the RUNX1 overexpression group exhibited a significant increase in lung weight and area of lung metastatic lesions, while mice in the RUNX1 knockdown group exhibited a reduction in lung weight and area of lung metastatic lesions **(Figures 3H, I)**. In summary, RUNX1 significantly enhanced liver and lung metastases in CRC models, highlighting its role in tumor aggressiveness.

**Figure 4:**
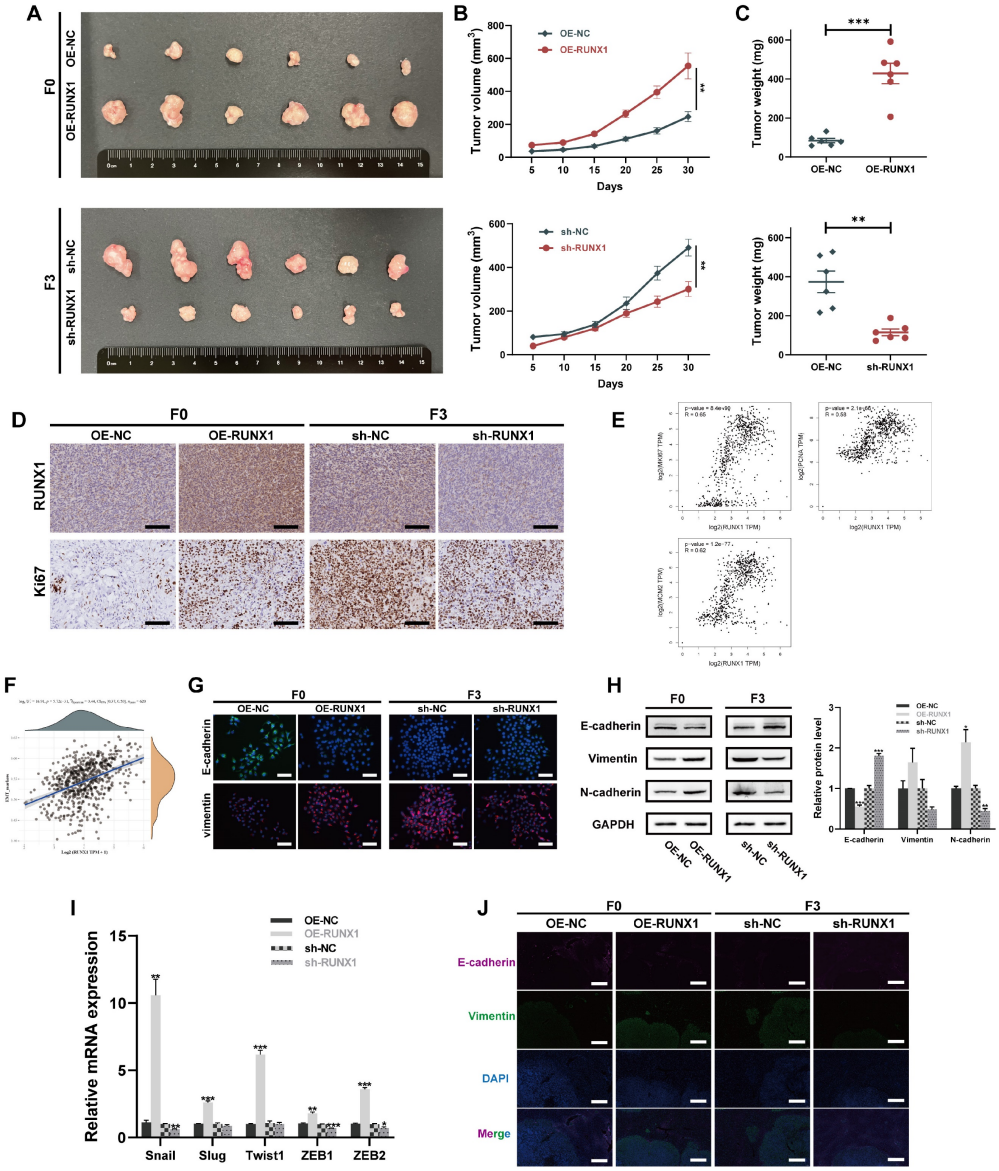
**RUNX1 enhances tumor growth and EMT in CRC. (A-C)** Representative graph of macroscopic overview of tumor xenografts, along with the growth curve depicting tumor volume and a measurement of tumor weight. **(D)** Representative images displaying RUNX1 and Ki67 staining in tumor xenografts from specified groups. Scale bar: 100 µm. **(E)** Correlation of RUNX1 with cell proliferation markers using Spearman's method (GEPIA database; R = Spearman coefficient). **(F)** ssGSEA analysis reveals a significant positive correlation between RUNX1 expression and EMT-associated markers, based on data from the TCGA database (Spearman ρ = 0.44, *P* = 5.72e-31). **(G)** Immunofluorescence staining illustrates RUNX1-mediated alterations in epithelial (E-cadherin) and mesenchymal (vimentin) markers in F0 (overexpression) and F3 (knockdown) CRC cells. Scale bar: 100 µm. **(H)** Western blot shows RUNX1 overexpression in F0 cells leads to EMT, evidenced by lowered E-cadherin and elevated N-cadherin and vimentin, while RUNX1 knockdown in F3 cells reverses this phenotype. Quantitative results are shown on the right. **(I)** qPCR findings demonstrated altered expression of EMT-related transcription factors (Snail, Slug, Twist1, ZEB1/2) consequent to RUNX1 manipulation in F0 and F3 cells. **(J)** Immunofluorescent staining in murine hepatic metastatic tissues for E-cadherin and Vimentin confirms RUNX1-induced EMT phenotype, corroborating *in vitro* findings. Scale bar: 400 µm. Error bars represent mean ± SEM of three independent experiments. *P < 0.05; **P < 0.01; ***P < 0.001.

To enhance the confirmation of the pro-carcinogenic effects of RUNX1 in an *in vivo* context, xenograft models were also established by subcutaneously injecting OE-RUNX1 (F0) or sh-RUNX1 (F3) cells (along with the respective negative control cells) into the flanks of nude mice. As illustrated in **Figures 4A-C**, RUNX1 downregulation led to decreased tumor volumes and weights, confirming that RUNX1 deficiency impeded tumor growth *in vivo*. Conversely, upon overexpression of RUNX1, both tumor volume and weight exhibited a notable increase in comparison to the control group. IHC staining of both RUNX1 and Ki67 revealed that the tumor xenografts with elevated RUNX1 expression exhibited notably enhanced proliferation capacities **(Figure 4D)**, implying a crucial function of RUNX1 in promoting the growth of CRC. Employing the GEPIA webserver for analysis of TCGA and GTEx databases, a positive correlation was identified between RUNX1 and cell proliferation markers (MKI67, PCNA, and MCM2) **(Figure 4E)**. These findings further reinforce our hypothesis connecting RUNX1 with the regulation of cell proliferation-related mechanisms in CRC progression.

### RUNX1 promotes EMT phenotype in CRC

Emerging evidence underscores the role of RUNX1 in promoting migration and invasion of CRC cells through the mechanism of epithelial-mesenchymal transition (EMT) 34. The prevailing consensus is that EMT serves as a crucial role in tumor progression, invasion, and metastasis, with cells undergoing EMT exhibiting increased metastatic potential and thus poorer survival rates 35. Given the indispensable contribution of EMT to the development and dissemination of tumors 36, a comprehensive analysis was undertaken to assess RUNX1's effects on EMT. ssGSEA, utilizing the TCGA database, indicated a statistically significant correlation positively linking RUNX1 expression with EMT-related markers (Spearman ρ = 0.44, *P* = 5.72e-31) **(Figure 4F)**. Immunofluorescence results showed that increased RUNX1 levels resulted in reduced expression of the epithelial marker E-cadherin and elevated expression of the mesenchymal marker vimentin in F0 cells. Conversely, reduced RUNX1 expression led to the opposite effects in F3 cells **(Figure 4G)**. In accordance with the immunofluorescent staining, western blot analysis corroborated diminished expression of E-cadherin, whereas elevated levels of N-cadherin and vimentin were observed. Conversely, sh-RUNX1 (F3) cells exhibited the opposite trend **(Figure 4H)**. Moreover, to further validate these observations, we employed qPCR assays to examine the expression levels of multiple EMT-associated transcription factors, including Snail, Slug, Twist1, and ZEB1/2. The data revealed an upregulation of these transcription factors in F0 cells with RUNX1 overexpression, while an inverse effect was observed in F3 cells upon RUNX1 knockdown **(Figure 4I)**. Consistent with these *in vitro* findings, immunofluorescence results from murine hepatic metastatic tissues further corroborated the notion that RUNX1 induces the EMT phenotype in colorectal cancer cells **(Figure 4J)**. The findings established RUNX1 as a significant promoter of EMT in colorectal cancer, directly implicating it in the enhancement of tumor invasiveness.

### RUNX1 regulation explored through ChIP-Seq and MUC13 target validation in CRC

Expanding on our previously published RNA-Seq findings that characterized the highly invasive phenotype of the F3-Caco-2 cell line 22, this study employed ChIP-Seq to identify direct transcriptional targets of RUNX1, aiming to further elucidate the genetic mechanisms underlying RUNX1's role in CRC aggressiveness. By integrating the ChIP-Seq data with the RNA-Seq dataset, the study aimed to pinpoint key genes that were not only transcriptionally upregulated in highly invasive F3 cells but also served as direct downstream targets of RUNX1. This integrative approach was designed to offer a comprehensive understanding of the mechanistic influence of RUNX1 on the invasive capabilities of colorectal cancer cells.

**Figure 5:**
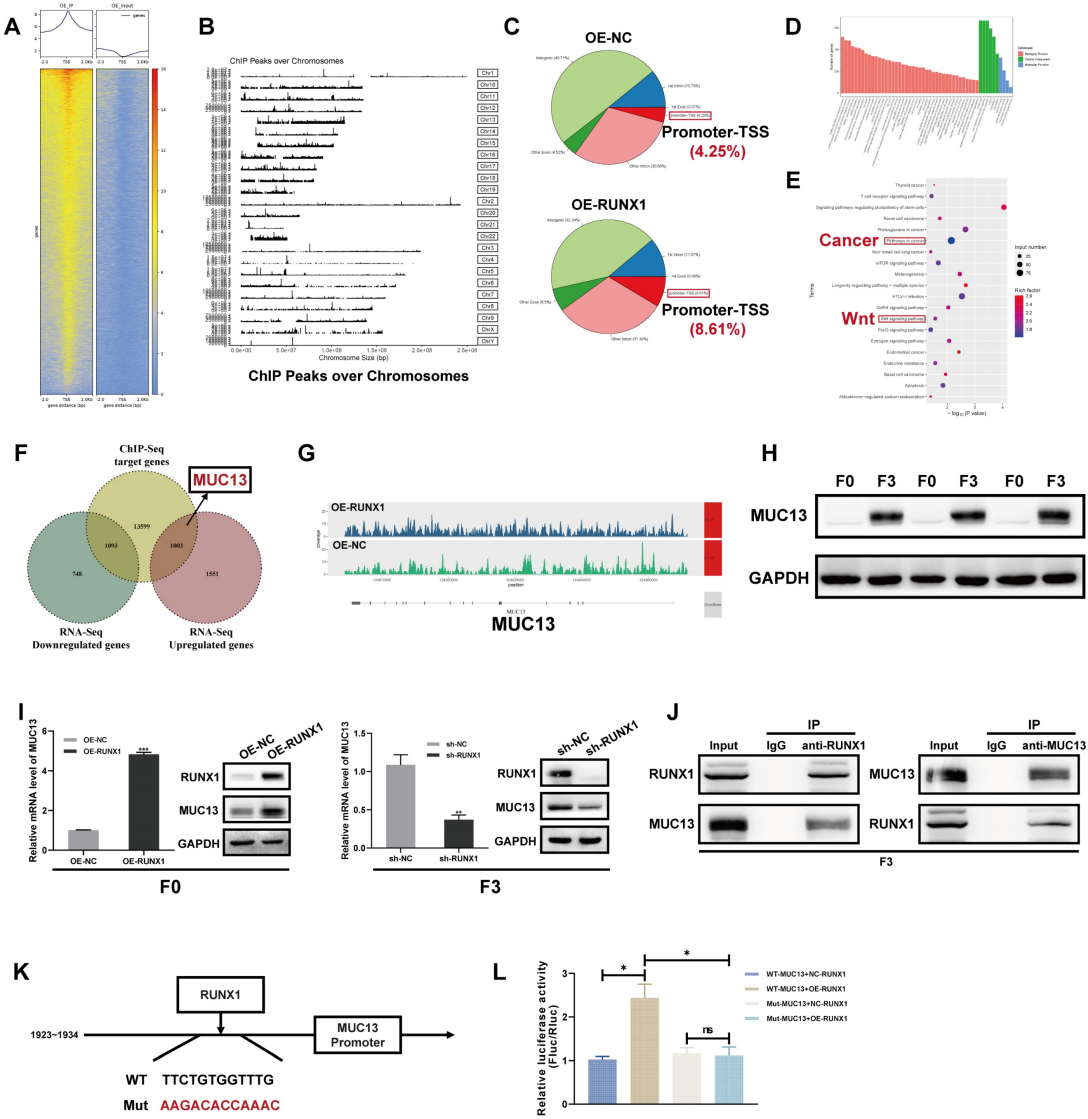
** ChIP-Seq identification of RUNX1 impact and validation of MUC13 in CRC. (A)** Heatmap displaying Input and IP Read counts near the transcription start site (TSS) in both OE-NC and OE-RUNX1 cell groups. **(B)** Visualization of the chromosomal locations of identified chromatin peaks associated with RUNX1 binding.** (C)** Bar graph depicting the percentage of peaks located in the promoter-TSS regions for OE-NC and OE-RUNX1 groups. **(D)** GO enrichment analysis results showing cellular functions predominantly influenced by RUNX1-targeted genes. **(E)** KEGG pathway analysis highlighting key cellular pathways influenced by RUNX1, such as cancer and stem cell pathways. **(F)** Venn diagram highlighting MUC13 as a shared target in ChIP-Seq and upregulated in RNA-Seq. **(G)** Differential distribution of MUC13-associated peaks in the RUNX1-overexpressing group compared to control cells. **(H)** Western blot confirmation of MUC13 expression in F0 and F3 cells, utilizing primary tumor cells from three different metastatic tissues for F3. **(I)** qPCR and Western blot validate MUC13 upregulation in RUNX1-overexpressing F0 cells. qPCR and Western blot confirm MUC13 downregulation following RUNX1 knockdown in F3 cells. **(J)** Co-IP assay confirms RUNX1-MUC13 interaction in F3 cells. **(K)** Depiction of wild-type and mutant MUC13 promoter constructs for luciferase assays. **(L)** Luciferase assays show enhanced activity with RUNX1 and the wild-type MUC13 promoter, while mutation reduces this effect, indicating the importance of RUNX1's binding site for MUC13 activation. ns, not significant; *P < 0.05; **P < 0.01; ***P < 0.001.

Consistent with the objective to identify RUNX1-regulated genes implicated in CRC progression, ChIP-Seq was utilized to scrutinize the binding patterns of RUNX1 in F0 cells. ChIP-Seq analyses revealed a total of 112,228 chromatin peaks linked to 12,830 genes, inclusive of MUC13, in both the OE-NC and OE-RUNX1 groups. A heat map of this data **(Figure 5A)** demonstrated complex Input and IP Read distribution near the TSS, with the IP group exhibiting significantly higher Read counts in the proximal TSS region compared to the Input group. The chromosomal locations of these peaks were further illustrated in **Figure 5B**. Subsequent in-depth genomic region-specific analysis indicated that 8.61% of peaks were located in the promoter-TSS area, with a higher prevalence observed in the OE-RUNX1 group compared to the OE-NC group (8.61% vs. 4.25%) **(Figure 5C)**. Statistically significant differential binding was observed in RUNX1-associated motifs, supported by notable *P-values* **(Supplementary Figure 4A)**.

Furthermore, interaction networks visualized the multifaceted relationships between motifs and transcription factors (TFs) **(Supplementary Figure 4B).** GO and KEGG enrichment analyses were conducted to identify functional roles and signaling pathways influenced by differential peaks between OE-NC and OE-RUNX1. GO analysis revealed that the genes targeted by RUNX1 were predominantly involved in cellular metabolic processes, mainly localized in the nucleus, and functionally implicated in transcription factor binding **(Figure 5D)**. KEGG analysis highlighted involvement in pathways such as ' signaling pathways regulating pluripotency of stem cells,' pathways in cancer,' ' mTOR signaling pathway,' ' GnRH signaling pathway,' and ' Wnt signaling pathway ' **(Figure 5E)**, implicating RUNX1's role in tumorigenesis.

A comprehensive examination of ChIP-Seq and RNA-Seq datasets identified MUC13 as a significant downstream target of RUNX1, notably in the highly invasive F3 cell line **(Figure 5F)**. The differential distribution of MUC13-associated peaks in the RUNX1-overexpressing group compared to controls was observed **(Figure 5G)**. These findings were corroborated via qPCR, confirming MUC13 as one of the most significantly upregulated genes in F3 cells **(Supplementary Figure 4D)**, in line with our previously published RNA-Seq findings 22 **(Supplementary Figure 4C)**. Strikingly pronounced differences in MUC13 expression between F0 and F3 cells were further substantiated through Western Blot analysis **(Figure 5H)**. Both qPCR and Western blot analyses corroborated the synchronous upregulation of MUC13 in RUNX1-overexpressing F0 cells and its downregulation following RUNX1 knockdown in F3 cells **(Figure 5I)**. Importantly, co-immunoprecipitation assays performed in F3 cells validated the protein-protein interaction between RUNX1 and MUC13 **(Figure 5J)**. Based on this collective evidence, we hypothesize that MUC13 serves as a downstream regulatory target of RUNX1, with their expression levels being correlated. Subsequently, dual-luciferase reporter assays were utilized to elucidate the transcriptional regulation of MUC13 by RUNX1, employing both the wild-type and mutated MUC13 promoter sequences to discern the specificity of the interaction. The wild-type promoter possessed the RUNX1 binding site at nucleotides 1923-1934, while the corresponding mutant variant was modified to impair this specific site **(Figure 5K)**. Luciferase activity assays indicated a significant increase with the co-transfection of RUNX1 and the MUC13 wild-type promoter, whereas activity from the mutant promoter exhibited a substantial decline, emphasizing the essential function of the RUNX1 binding site in MUC13 transcriptional activation **(Figure 5L)**. The experimental findings validate RUNX1's direct role in modulating MUC13 expression, emphasizing the critical importance of the binding site's sequence integrity for transcriptional activation and delineating the specificity of the RUNX1-MUC13 interaction as a pivotal modulator in CRC pathology.

### MUC13 overexpression and its significant positive correlation with RUNX1 as indicators of poor prognosis in colorectal cancer

**Figure 6:**
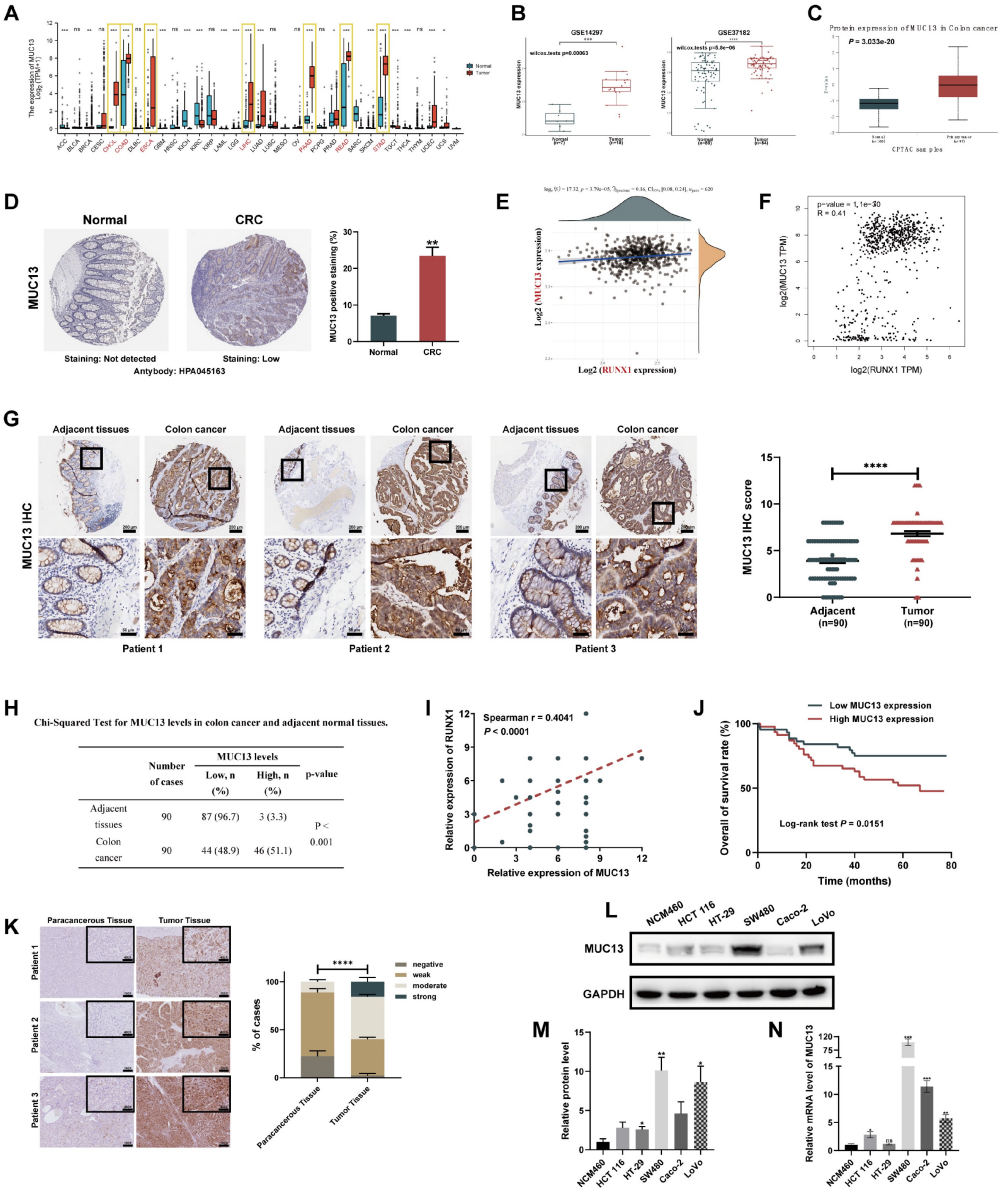
** Correlation between MUC13 and RUNX1 expression and its prognostic implications in colorectal cancer. (A)** TCGA data-based elevation of MUC13 expression across multiple gastrointestinal cancers. Statistically significant differences highlighted in red. **(B)** Upregulation of MUC13 in colorectal cancer tissues relative to normal controls, based on independent GEO datasets (GSE14297 and GSE37182), assessed using the Wilcoxon rank-sum test. **(C, D)** Protein-level confirmation of MUC13 overexpression in colorectal cancer tissues compared to normal controls, as validated by CPTAC (C) and HPA (D) databases. **(E, F)** Spearman correlation of RUNX1 and MUC13 expression using TCGA data (E) and GEPIA database (F), both revealing significant positive associations. **(G)** IHC staining of MUC13 in a TMA containing 90 CRC tumor and matched normal tissues (left). Quantitative analysis of MUC13 expression shown on the right, confirmed by unpaired t-test (P<0.0001). Scale bar: 200 μm and 50 μm, respectively. **(H)** Chi-square analysis reveals significantly elevated MUC13 expression in colorectal tumors compared to adjacent tissues (P<0.001). **(I)** Spearman analysis of MUC13 and RUNX1 protein levels using the same TMA reveals a significant positive correlation (Spearman r=0.4041, P<0.0001). **(J)** Kaplan-Meier analysis shows poorer prognosis for high MUC13 expression (Log-rank P=0.0151). **(K)** IHC staining reveals elevated MUC13 in hepatic metastatic lesions from 15 cases previously analyzed for RUNX1, quantified by two-way ANOVA (right). Scale bar: 200 μm (100X); 100 μm (400X). **(L)** Western blot analysis displaying MUC13 expression across five CRC cell lines and the NCM460 normal human colonic epithelial cell line. **(M)** Quantitative evaluation of MUC13 protein levels derived from Western blot data. **(N)** qPCR determination of MUC13 mRNA levels in the same cell lines as shown in (L)**.** Error bars represent mean ± SEM of three independent experiments. ns, not significant; *P < 0.05; **P < 0.01; ***P < 0.001; ****P < 0.0001.

To further evaluate the correlation between MUC13 and RUNX1, expression of MUC13 in various gastrointestinal tumors was initially assessed utilizing the TCGA database. Consistent with RUNX1 expression patterns, significant overexpression of MUC13 was observed in various gastrointestinal cancers, such as cholangiocarcinoma, colon cancer, esophageal cancer, hepatocellular carcinoma, pancreatic cancer, rectal cancer, and gastric cancer **(Figure 6A)**.

Additionally, two independent GEO microarray datasets (GSE14297 and GSE37182) were employed to assess the differential expression of MUC13 between normal and colorectal tumor tissues. Both datasets indicated significant upregulation of MUC13 in tumor samples as compared to normal controls **(Figure 6B)**. Protein-level validation, conducted via CPTAC and HPA databases, corroborated elevated MUC13 expression in tumor vs. normal tissues **(Figures 6C, D)**. Spearman analysis of TCGA data affirmed a significant positive association between RUNX1 and MUC13 expressions (**Figure 6E**; Spearman ρ=0.16, P=3.79e-05). This positive correlation was further supported by analyses in the GEPIA online database, which integrates samples from both TCGA and GTEx, and reached statistical significance (**Figure 6F**; R=0.41, P=1.1e-30). To substantiate the putative functional association and positive regulation between MUC13 and RUNX1 in colorectal cancer, IHC analyses were executed on a TMA previously employed for RUNX1 evaluations. As illustrated in **Figure 6G**, a significant upregulation of MUC13 was detected in tumor samples relative to matched normal ones, with an unpaired t-test confirming statistical significance (P<0.0001). Based on median expression levels, colorectal samples (n=90) were classified into low (n=44) and high (n=46) MUC13 expression groups, with Chi-square analyses revealing statistically significant high MUC13 expression in 51.1% of tumors compared to 3.3% in adjacent tissues (**Figure 6H**, *P*<0.001). Importantly, Spearman correlation analyses based on the same TMA revealed a significant positive correlation between the protein expression levels of MUC13 and RUNX1 (**Figure 6I**; Spearman r=0.4041, *P*<0.0001). As previously described, the 90 colorectal cancer patients in the TMA were stratified into two groups based on MUC13 expression levels.

Kaplan-Meier survival analysis revealed poorer prognoses for patients in the high MUC13 expression group **(Figure 6J)**. Building on this, Chi-square tests substantiated the significant association of high MUC13 expression with advanced AJCC stages (χ^2=13.098, *P*<0.001), higher T-stages (χ^2=8.855, *P*=0.003), and N-stages (χ^2=10.490, *P*=0.001), as well as poor clinical outcomes (χ^2=6.988, *P*=0.008) **(Table 2)**. Subsequently, IHC staining was applied to hepatic metastatic lesions from the same 15 cases previously analyzed for RUNX1. Marked upregulation of MUC13 was observed in these metastatic tissues compared to adjacent non-tumoral tissues **(Figure 6K)**. These findings are consistent with existing research on MUC13 37,38 and indicate a similar pattern of aberrant expression with RUNX1 in colorectal cancer. We next investigated MUC13 expression in colorectal cancer cell lines and found increased mRNA and protein levels compared to normal cells **(Figures 6L, M)**. Consistent with previous observations for RUNX1, a similar trend was observed in hepatocellular carcinoma cell lines **(Supplementary Figures 5A, B)**. In summary, the analysis established a significant positive correlation between MUC13 and RUNX1 expression levels, associated with poor prognosis in colorectal cancer.

### RUNX1 modulation and combined impact with MUC13 on *in vitro* colorectal cell behaviors

**Figure 7:**
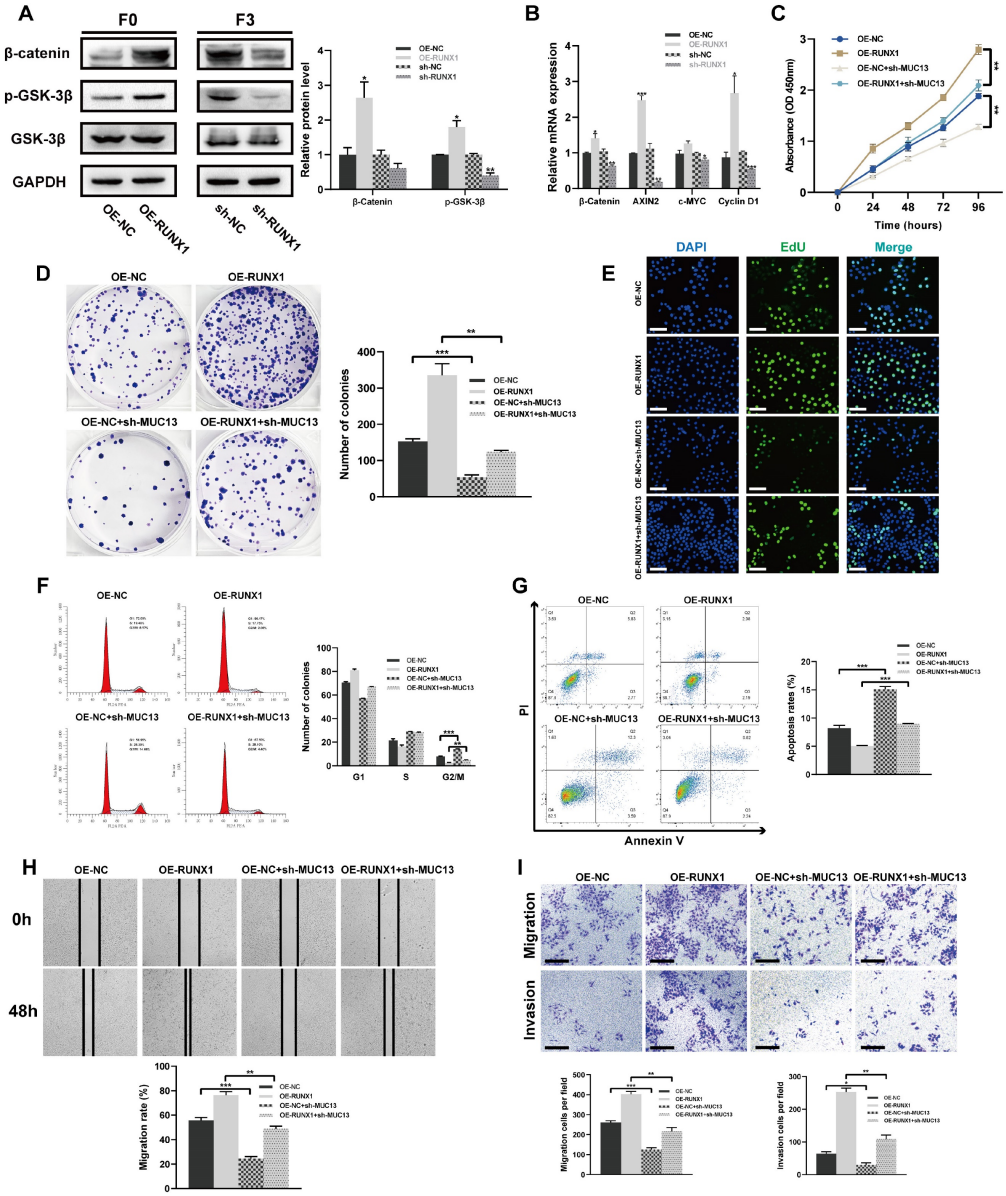
** Effects of RUNX1 modulation and MUC13 co-expression on colorectal cell properties *in vitro*. (A)** Western blot analyses showing changes in β-catenin, p-GSK-3β, and GSK-3β protein levels upon RUNX1 modulation in F0 and F3 cells. Quantitative analysis shown on the right. **(B)** qPCR results depicting alterations in mRNA levels of β-catenin, AXIN2, and Cyclin D1 upon RUNX1 modulation. **(C)** Proliferative effects of RUNX1 and MUC13 co-modulation assessed via CCK-8 assay. **(D)** Colony formation influenced by combined modulation of RUNX1 and MUC13. **(E)** Evaluation of cell proliferation using EdU assay upon concurrent modulation of RUNX1 and MUC13. Scale bar: 100 μm. **(F)** Cell cycle distribution alterations with RUNX1 and MUC13, analyzed through flow cytometry. **(G)** Annexin V-FITC/PI staining indicating the anti-apoptotic effects of RUNX1 and MUC13 co-modulation. **(H, I)** Scratch and Transwell migration and invasion assays served to investigate the effects of RUNX1 and MUC13 co-modulation on colorectal tumor cell motility. Scale bar: 100 μm. Quantification for (D, F, G, H, and I) represented in bar graphs. Error bars represent mean ± SEM of three independent experiments. *P < 0.05; **P < 0.01; ***P < 0.001.

β-catenin, a co-activator for LEF/TCF transcription factors, is pivotal in the Wnt signaling pathway 39. GSK3β regulates the pathway by controlling β-catenin degradation 39,40. Protein levels of p-GSK-3β, GSK-3β, and β-catenin were analyzed post-RUNX1 modulation. Overexpression of RUNX1 in F0 cells elevated β-catenin and p-GSK-3β levels, while its knockdown in F3 cells showed the reverse effect **(Figure 7A)**. Further, qPCR analysis revealed altered mRNA levels of key components upon RUNX1 modulation, with significant transcriptional changes observed in β-catenin, AXIN2, and Cyclin D1 **(Figure 7B)**. To elucidate the synergistic impact of RUNX1 and MUC13 on key cellular processes, F0 cells were co-transfected with lentiviruses for RUNX1 overexpression (OE-RUNX1) and MUC13 knockdown (sh-MUC13).

Cellular assays, including CCK-8, colony formation, and EdU, illustrated that the knockdown of MUC13 notably attenuated the proliferative advantage conferred by RUNX1 overexpression. Concurrently, cell cycle analysis revealed that MUC13 knockdown mitigated RUNX1-driven progression from G2/M to S phase **(Figures 7C-F)**. Annexin V-FITC/PI staining further indicated that this knockdown negated the anti-apoptotic effects of RUNX1 overexpression. Scratch-wound and Transwell assays demonstrated that MUC13 suppression reduced the enhanced migratory and invasive capabilities induced by RUNX1 **(Figures 7G-I)**. Overall, the results demonstrated that RUNX1 and MUC13 co-modulation distinctly influenced cellular behaviors related to colorectal cancer progression.

### MUC13's role in moderating *in vivo* effects of RUNX1 on colorectal tumor growth and metastasis

**Figure 8:**
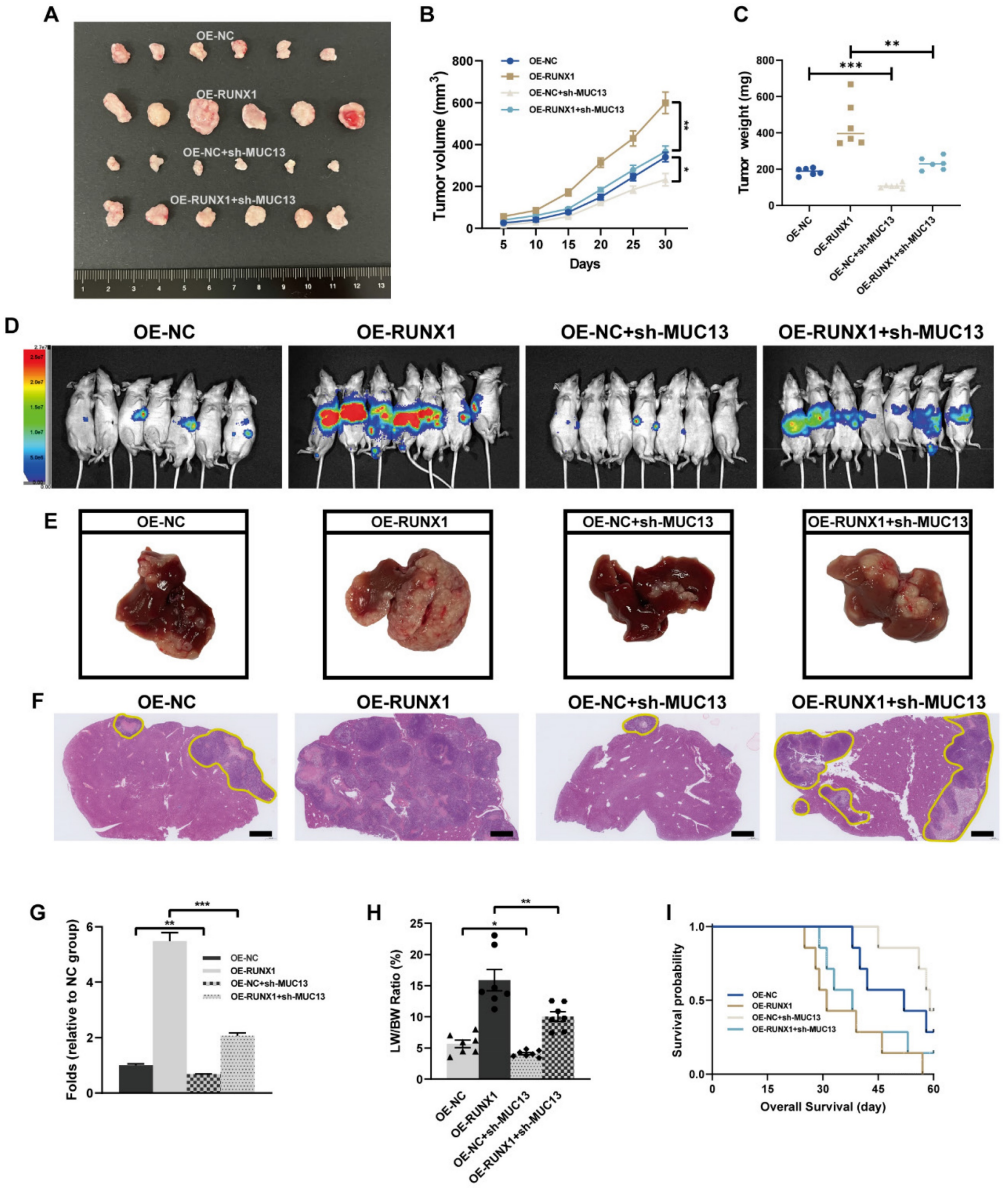
** MUC13 modulates the *in vivo* effects of RUNX1 on colorectal tumor growth and metastasis. (A)** Macroscopic assessment of subcutaneous tumor volumes 30 days post-inoculation reveals considerable enlargement in the RUNX1-overexpressing group, partially offset by MUC13 knockdown. **(B)** Tumor volume growth curve for RUNX1-overexpressing cells over 30 days, indicating a significant decrease upon MUC13 suppression. **(C)** Assessment of tumor weights highlighting the inhibitory impact of MUC13 downregulation on RUNX1-induced tumorigenesis. **(D)** Four weeks post-injection, *in vivo* BLI imaging reveals an enhanced liver metastasis signal from RUNX1 overexpression, partially alleviated by MUC13 knockdown. **(E)** Macroscopic images display the hepatic metastasis influenced by RUNX1 overexpression and MUC13 knockdown for the respective group. **(F)** Representative H&E-stained sections of liver tissue sections illustrate metastatic areas influenced by RUNX1 and MUC13 interactions. The highlighted areas, delineated with a yellow outline, exhibit an intensified purple color compared to the adjacent tissue. Scale bar: 1 mm. **(G)** Quantitative evaluation of H&E-stained sections. **(H)** Evaluation of the liver weight to body weight ratio (LW/BW) reveals an increase following RUNX1 overexpression, counteracted by MUC13 knockdown. **(I)** Kaplan-Meier survival curves reveal a possible enhancement in OS in mice with MUC13 knockdown, despite the elevated expression of RUNX1. The differences, as assessed by the log-rank test, did not achieve statistical significance. *P < 0.05; **P < 0.01; ***P < 0.001.

Given our *in vitro* findings, MUC13 knockdown counteracted the proliferative, migratory, and invasive effects induced by RUNX1 on colorectal cancer cells, prompting a deeper exploration of MUC13's role in RUNX1-driven *in vivo* tumor progression and metastasis. Subsequent to the subcutaneous inoculation of differentially transfected cell lines - OE-NC, OE-RUNX1, OE-NC+sh-MUC13, and OE-RUNX1+sh-MUC13 - into nude mice, a 30-day observation was conducted. Following the observation period, tumors were excised and subjected to macroscopic assessment. Tumors from the RUNX1-overexpressing group manifested a significant increase in volume, an effect partially offset by MUC13 knockdown, as illustrated in **Figure 8A**. Throughout the observation duration, the growth curve indicated a marked increase in tumor volume for the RUNX1-overexpressing cells relative to the control, an elevation considerably diminished upon MUC13 suppression **(Figure 8B)**. Similarly, upon quantifying tumor weights, the findings distinctly emphasized the inhibitory effect of MUC13 downregulation on the tumorigenic capabilities of RUNX1 overexpression **(Figure 8C)**.

After injecting cells from respective groups to the spleens of immunodeficient mice, an experimental model of liver metastasis model was developed. Four weeks post-injection, *in vivo* imaging of liver metastasis indicated that the BLI signal augmentation, attributed to RUNX1 overexpression, was partially reversed by MUC13 knockdown **(Figure 8D)**. Macroscopic and histological observations further confirmed the *in vivo* imaging results, confirming that MUC13 knockdown partially attenuated the pro-metastatic role of RUNX1 **(Figures 8E-G)**. Additionally, an elevation in the liver weight to body weight ratio (LW/BW) was observed following RUNX1 overexpression, which was subsequently attenuated by MUC13 knockdown **(Figure 8H)**. Kaplan-Meier survival curves revealed a potential mitigation of the diminished OS associated with RUNX1 overexpression by MUC13 knockdown, though without reaching statistical significance **(Figure 8I)**. IHC analysis of liver metastatic tissues indicated a positive relationship between the expression of MUC13 and RUNX1 proteins. Moreover, Ki67 staining revealed that co-transfection with OE-RUNX1 resulted in elevated colorectal cancer cell proliferation, an increase that MUC13 knockdown partially counteracted **(Supplementary Figure 6A).** Meanwhile, evaluation of TCGA and GTEx datasets via the GEPIA platform revealed a positive association between MUC13 and well-established cell proliferation markers, namely MKI67, MCM2, and PCNA** (Supplementary Figure 6B)**. Collectively, these findings indicated that MUC13 expression levels modulated the metastatic function of RUNX1 *in vivo*, suggesting that RUNX1 facilitated colorectal cancer cell metastasis partially via MUC13.

### RUNX1 and its influence on MUC13 in colorectal cancer mechanisms

**Figure 9:**
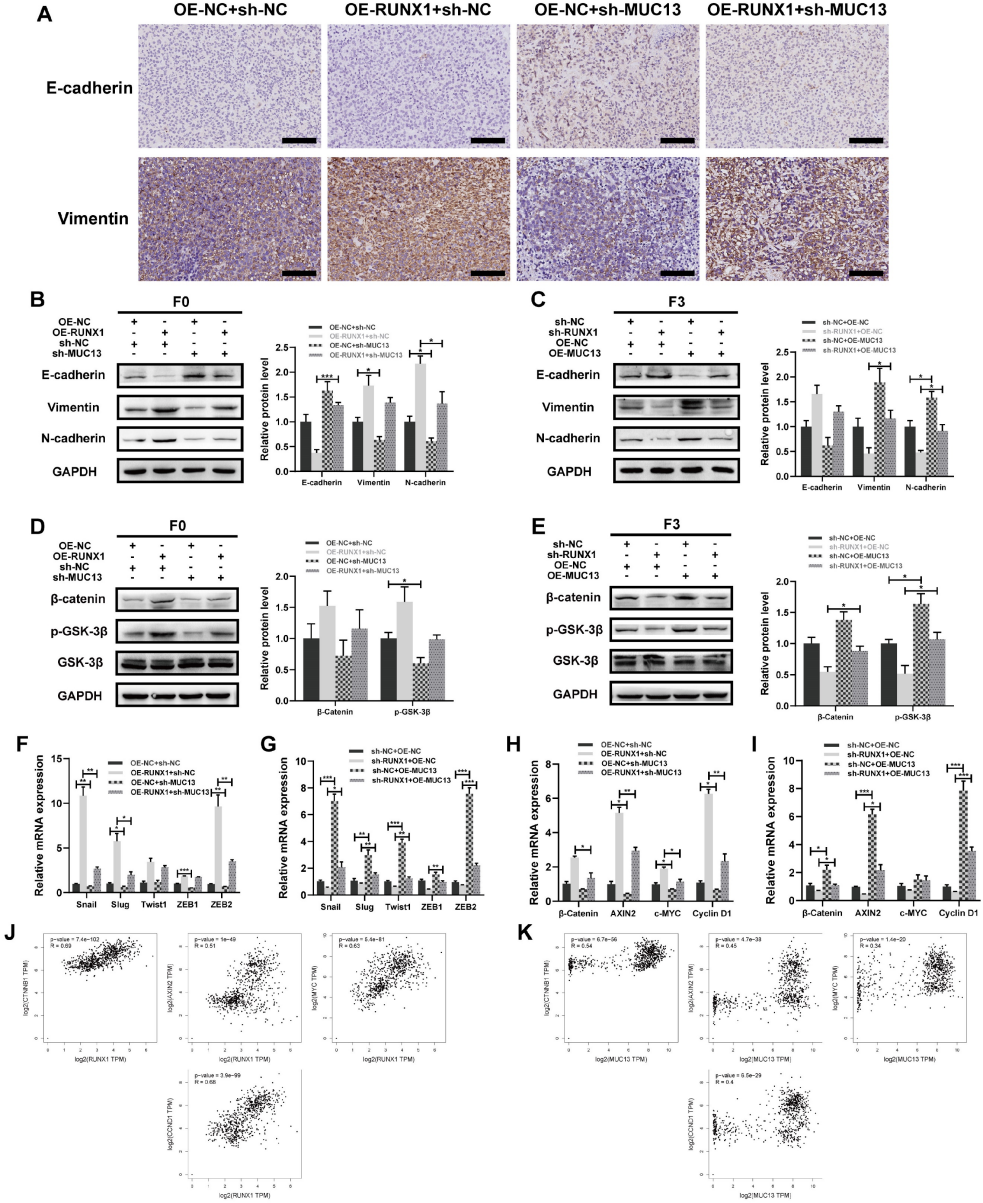
** Interactions and influence of RUNX1 and MUC13 on colorectal cancer mechanisms. (A)** The expression levels of E-cadherin and vimentin in murine liver metastatic tumor tissues were assessed through IHC staining. Scale bar: 100 μm. **(B, C)** Western blot analysis of EMT markers in F0 (B) and F3 (C) cells, illustrating effects of RUNX1 and MUC13 modifications. Accompanied by quantification of protein levels in bar graphs on the right. **(D, E)** Western blot analysis of the Wnt/β-catenin signaling pathway components (β-catenin, p-GSK-3β, GSK-3β) in F0 (D) and F3 (E) cells, with quantification in bar graphs on the right. **(F, G)** qPCR results of EMT-related transcription factors (Snail, Slug, Twist1, ZEB1, ZEB2) in F0 (F) and F3 (G) following RUNX1 and MUC13 modulation. **(H, I)** qPCR quantification of β-catenin, AXIN2, c-MYC, and Cyclin D1 transcripts in F0 (H) and F3 (I) cells, following genetic modulation of RUNX1 and MUC13. **(J, K)** Scatter plots derived from the GEPIA database illustrating correlations between RUNX1 or MUC13 expression and specified genes of the Wnt/β-catenin signaling pathway, based on Spearman correlation analysis. R, Spearman coefficient. Error bars represent mean ± SEM of three independent experiments. *P < 0.05; **P < 0.01; ***P < 0.001; ****P < 0.0001.

Numerous investigations have elucidated the significant association between the MUC gene family and EMT in tumor progression 41-44. Considering the critical role of EMT in tumor progression, invasion, and metastasis 35, this study investigated the regulatory function of RUNX1 in modulating MUC13 and its subsequent influence on the EMT phenotype in colorectal cancer cells. IHC staining revealed variations in EMT-associated markers in murine liver metastatic tumor tissues. Notably, knockdown of MUC13 was observed to oppose the EMT-inductive role of RUNX1, indicated by a modest upregulation of E-cadherin and a decrease in the mesenchymal marker, vimentin **(Figure 9A).** To elucidate the interactions between RUNX1 and MUC13, rescue assays were conducted in the context of F0 and F3 cell lines, employing lentivirus vectors for RUNX1 and MUC13 modulation, complemented with corresponding controls. Western blot analyses of F0 cells indicated that MUC13 knockdown effectively mitigated the EMT induced by RUNX1 overexpression. Conversely, in F3 cells, elevated MUC13 expression restored the EMT phenotype upon RUNX1 inhibition **(Figures 9B, C)**. Further supporting the Western blot results, qPCR analyses of EMT-transcription factors, including Snail, Slug, Twist1, and ZEB1/2, confirmed consistent findings **(Figures 9F, G)**. The findings suggested that RUNX1 modulated malignant properties in colorectal cancer cells, essentially through the transcriptional regulation of MUC13, which in turn affected EMT.

Aberrant activation of Wnt/β-catenin signaling serves as a distinctive hallmark of colorectal cancer, with mutations in this pathway being observed in 90% of advancing CRC tumors 45,46. Notably, the canonical Wnt/β-catenin pathway significantly influences the EMT process in tumors 47. RNA-Seq and ChIP-Seq KEGG analyses we conducted robustly emphasized the role of the Wnt pathway. From these observations, we inferred that RUNX1 and MUC13 might influence the malignancy of colorectal cancer via the Wnt pathway activity. To elucidate the potential relationship, Western Blotting was conducted to assess whether RUNX1 transcriptionally modulates MUC13, affecting the Wnt/β-catenin pathway activity. In F0 cells, MUC13 knockdown counteracted the activation of the Wnt/β-catenin pathway initiated by RUNX1 overexpression; conversely, enhanced MUC13 expression in F3 cells restored the pathway that was suppressed by RUNX1 knockdown **(Figures 9D, E)**. Additionally, qPCR analyses were conducted to evaluate the mRNA expression levels of critical molecules in the signaling pathway, specifically β-catenin, AXIN2, c-MYC, and Cyclin D1. The findings were consistent with the outcomes of the Western blotting **(Figures 9H, I)**. Subsequently, through the GEPIA platform with data derived from TCGA and GTEx datasets, the correlation between expression levels of RUNX1, MUC13, and specific molecules of the Wnt/β-catenin signaling pathway was explored. Evident correlations between RUNX1 and MUC13 with crucial molecules such as CTNNB1, AXIN2, MYC, and CCND1 were identified. Importantly, all observed associations consistently reached statistical significance, emphasizing the fundamental role of RUNX1 and MUC13 in modulating the Wnt/β-catenin signaling activities **(Figures 9J, K)**. Collectively, these findings partially substantiated the hypothesis that RUNX1 influenced the Wnt/β-catenin pathway through its interaction with MUC13.

### Modulation of Wnt/β-catenin signaling by RUNX1 and MUC13 through pharmacological interventions

**Figure 10:**
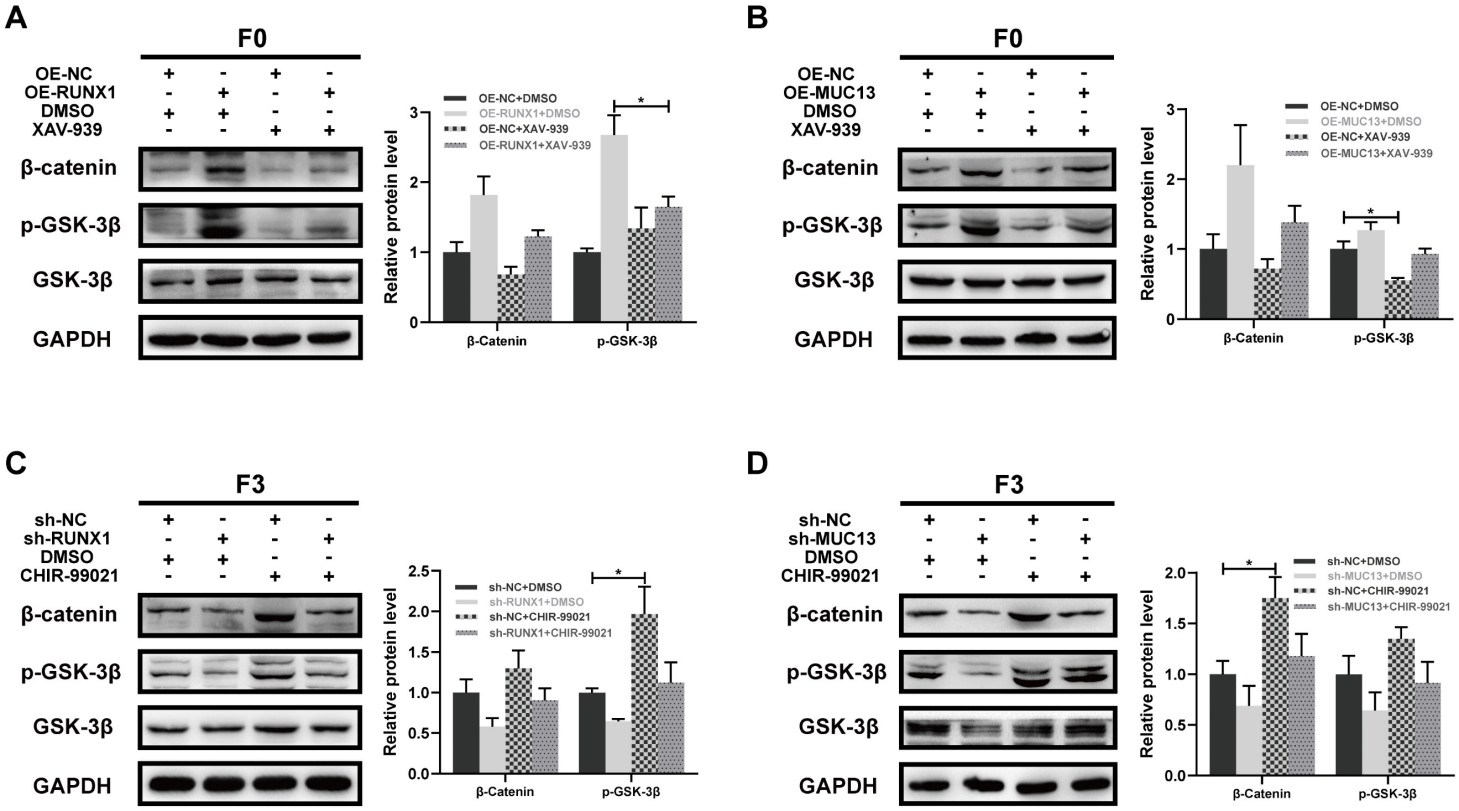
** Modulation of Wnt/**β**-catenin signaling by RUNX1 and MUC13 through pharmacological interventions. (A)** β-catenin and phosphorylated GSK-3β levels in F0 cells with RUNX1 overexpression treated with XAV-939. **(B)** β-catenin and phosphorylated GSK-3β levels in F0 cells with MUC13 overexpression treated with XAV-939. **(C)** β-catenin and phosphorylated GSK-3β levels in F3 cells with RUNX1 knockdown treated with CHIR-99021. **(D)** β-catenin and phosphorylated GSK-3β levels in F3 cells with MUC13 knockdown treated with CHIR-99021. Quantitative bar graphs are shown on the right of each panel. Error bars represent mean ± SEM of three independent experiments. *P < 0.05.

To further elucidate the role of RUNX1 and MUC13 in modulating the Wnt/β-catenin signaling pathway, we conducted experiments using the β-catenin-specific inhibitor XAV-939 and the GSK-3β-specific agonist CHIR-99021. In F0 cells with RUNX1 overexpression, treatment with XAV-939 significantly decreased β-catenin and phosphorylated GSK-3β levels compared to untreated cells **(Figure 10A)**. Similarly, in F0 cells overexpressing MUC13, XAV-939 treatment reduced β-catenin and phosphorylated GSK-3β levels **(Figure 10B)**. These results suggested that XAV-939 effectively disrupted the Wnt/β-catenin signaling pathway activated by RUNX1 and MUC13 overexpression. Conversely, in F3 cells with RUNX1 knockdown, treatment with CHIR-99021 partially restored β-catenin and phosphorylated GSK-3β levels **(Figure 10C)**. Similarly, in F3 cells with MUC13 knockdown, CHIR-99021 treatment partially reversed the decreased β-catenin and phosphorylated GSK-3β levels **(Figure 10D)**. These findings indicated that CHIR-99021 could reactivate the Wnt/β-catenin signaling pathway inhibited by RUNX1 and MUC13 knockdown. Overall, these experiments demonstrated that RUNX1 and MUC13 modulated β-catenin levels and activity through the Wnt/β-catenin signaling pathway. By utilizing specific inhibitors and activators, we highlighted the critical role of this pathway in mediating the effects of RUNX1 and MUC13 on β-catenin, providing deeper insights into the molecular mechanisms underlying CRC progression and potential therapeutic targets.

## Discussion

The latest 2022 data from the International Agency for Research on Cancer (IARC) positioned CRC as the third most frequently diagnosed cancer worldwide, with its mortality rates ascending to second place 1. As research on CRC metastasis advanced, the role of genetic interactions was emphasized as crucial to elucidating and controlling tumor progression. In our study, we focused on the significant impact of RUNX1 targeting MUC13 to facilitate metastasis. The identification of the RUNX1-MUC13 axis revealed a novel mechanism that potentially drives CRC metastasis through enhanced cell migration and invasion. Mechanistically, RUNX1 exerts direct regulation over MUC13, activating the Wnt/β-catenin pathway, a critical mediator in cancer progression and EMT. The clinical relevance of this pathway was underscored by both *in vitro* and *in vivo* validations, revealing that RUNX1 directly regulates MUC13, highlighting a potent target for potential therapeutic interventions intended for mitigating metastasis in CRC.

RUNX1, crucial in hematopoiesis, not only correlates with hematologic malignancies but also markedly influences solid tumors, as demonstrated by its elevated expression in epithelial cancers in the initial stages of tumor development 48. Further evidence of this is observed in the heightened expression levels of RUNX1 and its downstream target, REXO2, in isocitrate dehydrogenase wild-type low-grade gliomas, indicative of a poor prognosis 49.

Similarly, Liu *et al.* identified RUNX1 as an oncogenic contributor to pancreatic cancer growth, indicating its potential as both a prognostic biomarker and therapeutic target 12, supported by elevated RUNX1 RNA levels in cancerous compared to normal pancreatic tissues 50. Gao *et al.* elucidated in renal clear cell carcinoma that reduced RUNX1 promoter methylation correlates with higher expression and worse patient outcomes, underscoring its prognostic significance 51. Research conducted by Fernandez *et al.* substantiated that augmented expression of RUNX1 predominantly amplified the expression of cancer stem cell (CSC) markers in triple-negative breast cancer (TNBC) 52. Regarding angiogenesis, a pivotal process in oncogenesis 53, the impact of RUNX1 is substantial. In glioblastoma (GBM), knocking down RUNX1 in U-87 MG cells inhibited angiogenesis, with p38 MAPK pathway inhibition by SB203580 denoting RUNX1's involvement in promoting vascular growth via the p38 MAPK signaling pathway 54. RUNX1's pivotal role in modulating EMT and cellular stemness, which significantly related to the invasive characteristics of tumors, was critical for understanding its impact across different cancer types. In breast cancer, RUNX1 significantly influenced these cellular processes, enhancing tumor invasiveness 55. This modulation was similarly observed in cervical cancer, where elevated RUNX1 expression promoted EMT and markedly enhanced cancer cell invasion and metastasis 56. Additionally, elevated RUNX1 expression in prostate cancer related to the activation of Akt/P38/JNK-MAPK signaling pathways, contributing to enhanced EMT processes and increased metastatic potential 57. EMT's essential role in tumor progression, invasion, and metastasis is widely recognized. EMT-engaged cells typically display increased metastatic propensity, correlating with reduced survival outcomes 35. Due to its established involvement in aggressive tumor characteristics, RUNX1's functions in CRC require further investigation to understand its potential as a therapeutic target.

Abnormal expression of cell surface mucins, observed in a variety of tumors, correlates with the initiation, progression, and unfavorable prognosis of various adenocarcinomas 58, identifying mucin as a potential diagnostic biomarker and therapeutic target. Specifically, MUC13 is aberrantly expressed in different types of cancers and its functional mechanisms have been explored in multiple studies. In ovarian cancer, reduced methylation at CpG sites in MUC13's promoter resulted in overexpression, significantly enhancing the migratory and invasive abilities of cancer cells 59. Pang *et al.* confirmed that MUC13 contributes to the progression of lung cancer by enhancing the phosphorylation of ERK, JNK, and p38, thus activating the ERK signaling pathway 30. In digestive system cancer research, MUC13 was observed to be highly expressed in pancreatic cancer, impacting HER2 receptor tyrosine kinase activity, which initiated NF-κB p65 activation and nuclear translocation, along with IκB phosphorylation, thereby enhancing tumor progression 60. In gastric cancer, elevated MUC13 expression, driven by miR-212-3p and miR-132-3p, facilitated tumor progression 61. Particularly noteworthy is the research on MUC13 in colorectal cancer. MUC13 was demonstrated to enhance TNF-induced NF-κB activation, subsequently triggering the pathway and protecting colorectal cancer cells from apoptosis, with its high expression associated with tumor progression and metastasis 62. In a comprehensive study, Tripathi *et al.*, analyzing 196 colorectal cancer clinical samples, suggested MUC13 as a biomarker for initial detection and metastasis prediction, and an independent prognostic factor 63. All these findings were consistent with the conclusion that MUC13, regulated by RUNX1 transcription in colorectal cancer tissues, was associated with aggressive clinical features and poor prognosis. Investigations in this study revealed MUC13 as a downstream target of RUNX1, as identified through a comprehensive analysis that integrated RNA-Seq, ChIP-Seq, and bioinformatics. Subsequent validation, employing various online databases and tissue microarrays, demonstrated a significant elevation in MUC13 expression in colorectal cancer tissues compared to adjacent non-tumorous tissues. Importantly, the overexpression of MUC13 was consistently associated with more aggressive clinical features and a poorer prognosis. Moreover, a significant positive correlation between the expressions of MUC13 and RUNX1 in colorectal cancer tissues provided new insights into RUNX1's role in promoting malignant behavior in colorectal cancer.

It is well-established that the classic Wnt/β-catenin pathway plays a crucial role in various oncological processes, particularly in colorectal cancer, by promoting cell proliferation and inhibiting apoptosis 64. Dysregulated activation of the Wnt/β-catenin pathway is a known hallmark in the initiation of colorectal cancer 45. Mutations in the Wnt/β-catenin pathway, identified in around 90% of colorectal cancer cases 46,65, have been associated with the development of treatment resistance 66. These genetic alterations affect cellular functions including proliferation, apoptosis, and autophagy, and influence tumor behavior by altering metabolic regulation, immune responses, and EMT induction, critical for tumor heterogeneity and metastasis progression 67,68. The relationship between the Wnt/β-catenin pathway, RUNX1, and MUC13, notably the mucin family's MUC13, has been conclusively demonstrated, especially regarding gastrointestinal tumors 69. RUNX1 is known to activate the Wnt/β-catenin pathway in leukemia, facilitating the transformation of malignant stem cells 70. Similarly, MUC13 has been demonstrated to activate the Wnt signaling pathway in hepatocellular carcinoma, promoting tumor progression through β-catenin activation 25,31. The most compelling evidence for the importance of the Wnt signaling pathway in this context comes from RNA-Seq and ChIP-Seq analyses, which revealed significant enrichment in the KEGG pathways related to Wnt signaling. Based on these findings, it was hypothesized that RUNX1 and MUC13 could influence the malignant phenotype of colorectal cancer by modulating the activity of the Wnt pathway. This research aimed to validate this hypothesis by focusing on the novel regulatory relationship between RUNX1 and MUC13, investigating the impact of manipulating their gene expression on CRC malignancy in both *in vivo* and *in vitro* models, and assessing the resultant activity changes in the Wnt signaling pathway. The results demonstrated the pivotal role of the interaction between the transcription factor RUNX1 and MUC13 in modulating colorectal cancer malignancy via the Wnt pathway. Alterations in RUNX1 and MUC13 expression significantly impacted the classic Wnt/β-catenin signaling pathway, affecting crucial oncogenic processes, including cellular proliferation and EMT. Notably, modulation of MUC13 expression mitigated RUNX1's oncogenic effects, primarily by reducing EMT characteristics. Furthermore, RUNX1 enhanced the progression and metastatic capabilities of colorectal cancer by upregulating MUC13, which resulted in an increased EMT phenotype and amplified Wnt/β-catenin pathway activity. These insights significantly contribute to our understanding of the novel interactions between RUNX1 and MUC13 and their substantial impact on Wnt/β-catenin signaling in colorectal cancer.

There are some limitations in our research. Firstly, the current study relies predominantly on IHC for analyzing clinical samples. However, complementary validations through qPCR and Western blot, while crucial, are not conducted due to limitations tied to the application of TMA and histological sections. Secondly, although the conclusions regarding RUNX1's transcriptional regulation of MUC13 are supported by both Co-IP and dual-luciferase reporter assays, further validations are necessary to fully confirm these findings. Finally, while this research focuses specifically on the interaction between RUNX1 and MUC13, the partial recovery observed upon MUC13 knockdown suggests that additional, MUC13-independent mechanisms contribute to the maintenance of these phenotypes, highlighting the complexity of CRC pathogenesis and the necessity for further investigation into overlapping or compensatory pathways. Despite inherent limitations, the study validated the integral role of the RUNX1-MUC13 axis in the progression of colorectal cancer and promoted further investigations into targeted therapeutic strategies that have the potential to innovate colorectal cancer treatment.

## Conclusions

**Figure 11:**
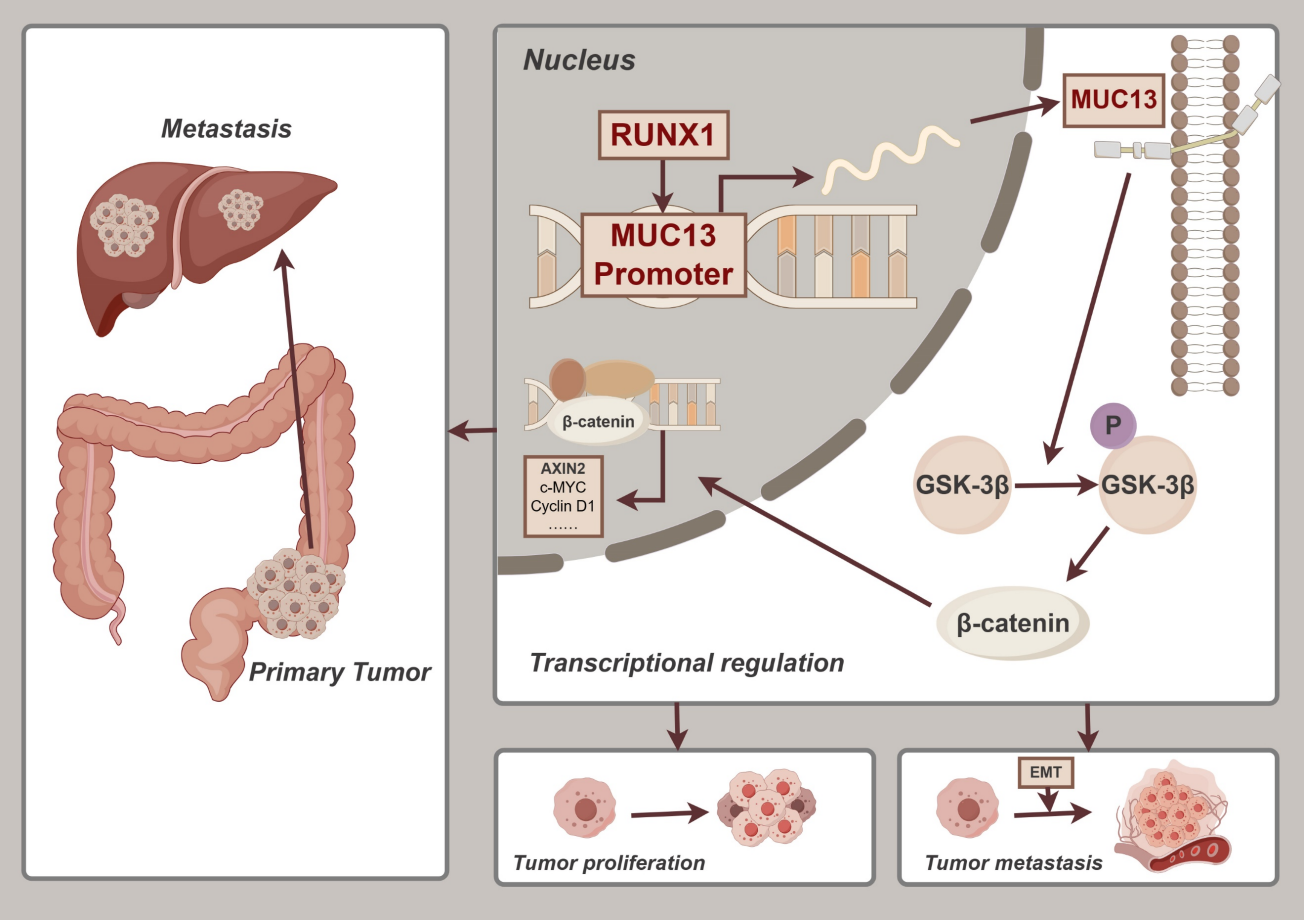
** RUNX1-MUC13 regulatory mechanisms in CRC metastasis.** The schematic demonstrates RUNX1's activation of MUC13 transcription within the cell nucleus, consequently amplifying Wnt/β-catenin pathway signaling. This activation initiates a succession of biological processes facilitating CRC proliferation and the advancement of EMT, leading to liver metastasis.

In conclusion, this study elucidated a pivotal RUNX1-MUC13 axis modulating the pathogenesis of CRC via the Wnt/β-catenin pathway. Enhanced RUNX1 expression was associated with increased malignancy and adverse prognostic outcomes in CRC, partly attributable to its regulatory effect on MUC13, which consequently amplified the EMT and metastatic capacity. Notably, this study identifies RUNX1 as a direct regulator of MUC13, which significantly enhances our understanding of the molecular mechanisms involved in CRC. The elucidation of the RUNX1-MUC13 interaction contributes to the identification of these molecules as potential biomarkers and therapeutic targets, considering their significant role in tumor invasiveness and dissemination **(Figure 11)**. The research enhances comprehension of molecular mechanisms involved in CRC development and facilitates the identification of innovative therapeutic approaches to decelerate this disease's progression.

## Supplementary Material

Supplementary figures and tables.

## Figures and Tables

**Table 1:** Correlation of RUNX1 expression with clinicopathological features in CRC.

Characteristic	Number	RUNX1 levels		χ^2^	p-value
		Low, n (%)	High, n (%)		
Gender					
Male	47	22 (46.8)	25 (53.2)	0.401	0.527
Female	43	23 (53.5)	20 (46.5)		
Age, years					
>65	44	22 (50.0)	22 (50.0)	0.000	1.000
≤65	46	23 (50.0)	23 (50.0)		
Grade					
G1-2	77	43 (55.8)	34 (44.2)	7.283	0.007 **
G3-4	13	2 (15.4)	11 (84.6)		
AJCC Stage					
Stage I-II	59	37 (62.7)	22 (37.3)	11.072	<0.001 ***
Stage III-IV	31	8 (25.8)	23 (74.2)		
T stage					
T1-2	11	11 (100.0)	0 (0.0)	12.532	<0.001 ***
T3-4	79	34 (43.0)	45 (57.0)		
N stage					
N0	61	37 (60.7)	24 (39.3)	8.598	0.003 **
N1-2	29	8 (27.6)	21 (72.4)		
M stage					
M0	87	45 (51.7)	42 (48.3)	3.103	0.242
M1	3	0 (0.0)	3 (100.0)		
Clinical outcome					
Alive	55	33 (60.0)	22 (40.0)	5.657	0.017 *
Dead	35	12 (34.3)	23 (65.7)		

*p < 0.05, **p < 0.01, ***p < 0.001.

**Table 2:** Correlation of MUC13 expression with clinicopathological features in CRC.

Characteristic	Number	MUC13 levels		χ^2^	p-value
		Low, n (%)	High, n (%)		
Gender					
Male	47	20 (42.6)	27 (57.4)	1.580	0.209
Female	43	24 (55.8)	19 (44.2)		
Age, years					
>65	44	21 (47.7)	23 (52.3)	0.047	0.829
≤65	46	23 (50.0)	23 (50.0)		
Grade					
G1-2	77	40 (51.9)	37 (48.1)	1.997	0.158
G3-4	13	4 (30.8)	9 (69.2)		
AJCC Stage					
Stage I-II	59	37 (62.7)	22 (37.3)	13.098	<0.001 ***
Stage III-IV	31	7 (22.6)	24 (77.4)		
T stage					
T1-2	11	10 (90.9)	1 (9.1)	8.855	0.003 **
T3-4	79	34 (43.0)	45 (57.0)		
N stage					
N0	61	37 (60.7)	24 (39.3)	10.490	0.001 **
N1-2	29	7 (24.1)	22 (75.9)		
M stage					
M0	87	43 (49.4)	44 (50.6)	0.301	1
M1	3	1 (33.3)	2 (66.7)		
Clinical outcome					
Alive	55	33 (60.0)	22 (40.0)	6.988	0.008 **
Dead	35	11 (31.4)	24 (68.6)		

**p < 0.01, ***p < 0.001.

## Abbreviations

RUNX1: Runt-related transcription factor 1
MUC13: Mucin 13
CRC: Colorectal cancer
EMT: Epithelial-mesenchymal transition
ChIP-seq: Chromatin immunoprecipitation sequencing
RNA-seq: RNA sequencing
CRLM: Colorectal liver metastasis
TMA: Tissue microarray
TCGA: The Cancer Genome Atlas
GEO: Gene Expression Omnibus
TIMER: Tumor Immune Estimation Resource
GEPIA: Gene Expression Profiling Interactive Analysis
HPA: Human Protein Atlas
CPTAC: Clinical Proteomic Tumor Analysis Consortium
Co-IP: Co-immunoprecipitation
BLI: Bioluminescence imaging
LW/BW: Liver weight to body weight ratio
AJCC: American Joint Committee on Cancer
ssGSEA: Single-sample gene set enrichment analysis
TSS: Transcription start site

## References

[1] Bray F, Laversanne M, Sung H, Ferlay J, Siegel RL, Soerjomataram I (2024). Global cancer statistics 2022: GLOBOCAN estimates of incidence and mortality worldwide for 36 cancers in 185 countries. CA Cancer J Clin.

[2] Fakih MG (2015). Metastatic colorectal cancer: current state and future directions. J Clin Oncol.

[3] Bhullar DS, Barriuso J, Mullamitha S, Saunders MP, O'Dwyer ST, Aziz O (2019). Biomarker concordance between primary colorectal cancer and its metastases. EBioMedicine.

[4] Stangl R, Altendorf-Hofmann A, Charnley RM, Scheele J (1994). Factors influencing the natural history of colorectal liver metastases. Lancet.

[5] Levanon D, Groner Y (2004). Structure and regulated expression of mammalian RUNX genes. Oncogene.

[6] Ito Y, Bae SC, Chuang LS (2015). The RUNX family: developmental regulators in cancer. Nat Rev Cancer.

[7] Sood R, Kamikubo Y, Liu P (2017). Role of RUNX1 in hematological malignancies. Blood.

[8] Keita M, Bachvarova M, Morin C, Plante M, Gregoire J, Renaud MC (2013). The RUNX1 transcription factor is expressed in serous epithelial ovarian carcinoma and contributes to cell proliferation, migration and invasion. Cell Cycle.

[9] Fu Y, Sun S, Man X, Kong C (2019). Increased expression of RUNX1 in clear cell renal cell carcinoma predicts poor prognosis. PeerJ.

[10] Teng H, Wang P, Xue Y, Liu X, Ma J, Cai H (2016). Role of HCP5-miR-139-RUNX1 Feedback Loop in Regulating Malignant Behavior of Glioma Cells. Mol Ther.

[11] Mitsuda Y, Morita K, Kashiwazaki G, Taniguchi J, Bando T, Obara M (2018). RUNX1 positively regulates the ErbB2/HER2 signaling pathway through modulating SOS1 expression in gastric cancer cells. Sci Rep.

[12] Liu S, Xie F, Gan L, Peng T, Xu X, Guo S (2020). Integration of transcriptome and cistrome analysis identifies RUNX1-target genes involved in pancreatic cancer proliferation. Genomics.

[13] Ramaswamy S, Ross KN, Lander ES, Golub TR (2003). A molecular signature of metastasis in primary solid tumors. Nat Genet.

[14] Fritz AJ, Hong D, Boyd J, Kost J, Finstaad KH, Fitzgerald MP (2020). RUNX1 and RUNX2 transcription factors function in opposing roles to regulate breast cancer stem cells. J Cell Physiol.

[15] Doll A, Gonzalez M, Abal M, Llaurado M, Rigau M, Colas E (2009). An orthotopic endometrial cancer mouse model demonstrates a role for RUNX1 in distant metastasis. Int J Cancer.

[16] Wang T, Jin H, Hu J, Li X, Ruan H, Xu H (2020). COL4A1 promotes the growth and metastasis of hepatocellular carcinoma cells by activating FAK-Src signaling. J Exp Clin Cancer Res.

[17] van Bragt MP, Hu X, Xie Y, Li Z (2014). RUNX1, a transcription factor mutated in breast cancer, controls the fate of ER-positive mammary luminal cells. Elife.

[18] Chimge NO, Little GH, Baniwal SK, Adisetiyo H, Xie Y, Zhang T (2016). RUNX1 prevents oestrogen-mediated AXIN1 suppression and beta-catenin activation in ER-positive breast cancer. Nat Commun.

[19] Bridges K, Yao HH, Nicol B (2022). Loss of Runx1 Induces Granulosa Cell Defects and Development of Ovarian Tumors in the Mouse. Int J Mol Sci.

[20] Lu C, Yang Z, Yu D, Lin J, Cai W (2020). RUNX1 regulates TGF-beta induced migration and EMT in colorectal cancer. Pathol Res Pract.

[21] Fijneman RJ, Anderson RA, Richards E, Liu J, Tijssen M, Meijer GA (2012). Runx1 is a tumor suppressor gene in the mouse gastrointestinal tract. Cancer Sci.

[22] Chen X, Tu J, Liu C, Wang L, Yuan X (2022). MicroRNA-621 functions as a metastasis suppressor in colorectal cancer by directly targeting LEF1 and suppressing Wnt/beta-catenin signaling. Life Sci.

[23] McGuckin MA, Linden SK, Sutton P, Florin TH (2011). Mucin dynamics and enteric pathogens. Nat Rev Microbiol.

[24] Sheng YH, Lourie R, Linden SK, Jeffery PL, Roche D, Tran TV (2011). The MUC13 cell-surface mucin protects against intestinal inflammation by inhibiting epithelial cell apoptosis. Gut.

[25] Dai Y, Liu L, Zeng T, Liang JZ, Song Y, Chen K (2018). Overexpression of MUC13, a Poor Prognostic Predictor, Promotes Cell Growth by Activating Wnt Signaling in Hepatocellular Carcinoma. Am J Pathol.

[26] Shimamura T, Ito H, Shibahara J, Watanabe A, Hippo Y, Taniguchi H (2005). Overexpression of MUC13 is associated with intestinal-type gastric cancer. Cancer Sci.

[27] Chauhan SC, Ebeling MC, Maher DM, Koch MD, Watanabe A, Aburatani H (2012). MUC13 mucin augments pancreatic tumorigenesis. Mol Cancer Ther.

[28] Sheng Y, Ng CP, Lourie R, Shah ET, He Y, Wong KY (2017). MUC13 overexpression in renal cell carcinoma plays a central role in tumor progression and drug resistance. Int J Cancer.

[29] Chauhan SC, Vannatta K, Ebeling MC, Vinayek N, Watanabe A, Pandey KK (2009). Expression and functions of transmembrane mucin MUC13 in ovarian cancer. Cancer Res.

[30] Pang Y, Zhang Y, Zhang HY, Wang WH, Jin G, Liu JW (2022). MUC13 promotes lung cancer development and progression by activating ERK signaling. Oncol Lett.

[31] Kang Q, Tingting W, Bingzi D, Hao Z, Yuwei X, Chuandong S (2024). GCNT3 regulated MUC13 to promote the development of hepatocellular carcinoma through the GSK3beta/beta-catenin pathway. Dig Liver Dis.

[32] Siegel RL, Miller KD, Fuchs HE, Jemal A (2022). Cancer statistics, 2022. CA Cancer J Clin.

[33] Manfredi S, Lepage C, Hatem C, Coatmeur O, Faivre J, Bouvier AM (2006). Epidemiology and management of liver metastases from colorectal cancer. Ann Surg.

[34] Li Q, Lai Q, He C, Fang Y, Yan Q, Zhang Y (2019). RUNX1 promotes tumour metastasis by activating the Wnt/beta-catenin signalling pathway and EMT in colorectal cancer. J Exp Clin Cancer Res.

[35] Karlsson MC, Gonzalez SF, Welin J, Fuxe J (2017). Epithelial-mesenchymal transition in cancer metastasis through the lymphatic system. Mol Oncol.

[36] Smith BN, Bhowmick NA (2016). Role of EMT in Metastasis and Therapy Resistance. J Clin Med.

[37] Sheng YH, Wong KY, Seim I, Wang R, He Y, Wu A (2019). MUC13 promotes the development of colitis-associated colorectal tumors via beta-catenin activity. Oncogene.

[38] Gupta BK, Maher DM, Ebeling MC, Stephenson PD, Puumala SE, Koch MR (2014). Functions and regulation of MUC13 mucin in colon cancer cells. J Gastroenterol.

[39] Stewart DJ, Chang DW, Ye Y, Spitz M, Lu C, Shu X (2014). Wnt signaling pathway pharmacogenetics in non-small cell lung cancer. Pharmacogenomics J.

[40] Taurin S, Sandbo N, Qin Y, Browning D, Dulin NO (2006). Phosphorylation of beta-catenin by cyclic AMP-dependent protein kinase. J Biol Chem.

[41] Ponnusamy MP, Lakshmanan I, Jain M, Das S, Chakraborty S, Dey P (2010). MUC4 mucin-induced epithelial to mesenchymal transition: a novel mechanism for metastasis of human ovarian cancer cells. Oncogene.

[42] Gnemmi V, Bouillez A, Gaudelot K, Hemon B, Ringot B, Pottier N (2014). MUC1 drives epithelial-mesenchymal transition in renal carcinoma through Wnt/beta-catenin pathway and interaction with SNAIL promoter. Cancer Lett.

[43] Lin H, Yang B, Teng M (2017). T-cell immunoglobulin mucin-3 as a potential inducer of the epithelial-mesenchymal transition in hepatocellular carcinoma. Oncol Lett.

[44] Marimuthu S, Rauth S, Ganguly K, Zhang C, Lakshmanan I, Batra SK (2021). Mucins reprogram stemness, metabolism and promote chemoresistance during cancer progression. Cancer Metastasis Rev.

[45] Najdi R, Holcombe RF, Waterman ML (2011). Wnt signaling and colon carcinogenesis: beyond APC. J Carcinog.

[46] Giles RH, van Es JH, Clevers H (2003). Caught up in a Wnt storm: Wnt signaling in cancer. Biochim Biophys Acta.

[47] Liu CC, Cai DL, Sun F, Wu ZH, Yue B, Zhao SL (2017). FERMT1 mediates epithelial-mesenchymal transition to promote colon cancer metastasis via modulation of beta-catenin transcriptional activity. Oncogene.

[48] Scheitz CJ, Lee TS, McDermitt DJ, Tumbar T (2012). Defining a tissue stem cell-driven Runx1/Stat3 signalling axis in epithelial cancer. EMBO J.

[49] Wang H, Wang X, Xu L, Zhang J, Cao H (2021). RUNX1 and REXO2 are associated with the heterogeneity and prognosis of IDH wild type lower grade glioma. Sci Rep.

[50] Liu S, Zhang J, Yin L, Wang X, Zheng Y, Zhang Y (2020). The lncRNA RUNX1-IT1 regulates C-FOS transcription by interacting with RUNX1 in the process of pancreatic cancer proliferation, migration and invasion. Cell Death Dis.

[51] Gao K, Zhang F, Chen K, Li W, Guan YB, Xu ML (2021). Expression patterns and prognostic value of RUNX genes in kidney cancer. Sci Rep.

[52] Fernandez NB, Sosa SM, Roberts JT, Recouvreux MS, Rocha-Viegas L, Christenson JL (2023). RUNX1 Is Regulated by Androgen Receptor to Promote Cancer Stem Markers and Chemotherapy Resistance in Triple Negative Breast Cancer. Cells.

[53] Yu X, Ye F (2020). Role of Angiopoietins in Development of Cancer and Neoplasia Associated with Viral Infection. Cells.

[54] Sangpairoj K, Vivithanaporn P, Apisawetakan S, Chongthammakun S, Sobhon P, Chaithirayanon K (2017). RUNX1 Regulates Migration, Invasion, and Angiogenesis via p38 MAPK Pathway in Human Glioblastoma. Cell Mol Neurobiol.

[55] Khawaled S, Aqeilan RI (2017). RUNX1, a new regulator of EMT in breast cancer. Oncotarget.

[56] Zheng LL, Cai L, Zhang XQ, Lei Z, Yi CS, Liu XD (2022). Dysregulated RUNX1 Predicts Poor Prognosis by Mediating Epithelialmesenchymal Transition in Cervical Cancer. Curr Med Sci.

[57] Janta S, Pranweerapaiboon K, Vivithanaporn P, Plubrukarn A, Chairoungdua A, Prasertsuksri P (2023). Holothurin A Inhibits RUNX1-Enhanced EMT in Metastasis Prostate Cancer via the Akt/JNK and P38 MAPK Signaling Pathway. Mar Drugs.

[58] Jonckheere N, Skrypek N, Van Seuningen I (2014). Mucins and tumor resistance to chemotherapeutic drugs. Biochim Biophys Acta.

[59] Sung HY, Park AK, Ju W, Ahn JH (2014). Overexpression of mucin 13 due to promoter methylation promotes aggressive behavior in ovarian cancer cells. Yonsei Med J.

[60] Kumari S, Khan S, Gupta SC, Kashyap VK, Yallapu MM, Chauhan SC (2018). MUC13 contributes to rewiring of glucose metabolism in pancreatic cancer. Oncogenesis.

[61] He L, Qu L, Wei L, Chen Y, Suo J (2017). Reduction of miR-132-3p contributes to gastric cancer proliferation by targeting MUC13. Mol Med Rep.

[62] Sheng YH, He Y, Hasnain SZ, Wang R, Tong H, Clarke DT (2017). MUC13 protects colorectal cancer cells from death by activating the NF-kappaB pathway and is a potential therapeutic target. Oncogene.

[63] Tripathi MK, Zacheaus C, Doxtater K, Stiles Z, Keramatnia F, Zafar N (2018). MUC13 is a novel molecular signature, for early detection and metastatic colorectal cancer. Cancer Res.

[64] Yu F, Yu C, Li F, Zuo Y, Wang Y, Yao L (2021). Wnt/beta-catenin signaling in cancers and targeted therapies. Signal Transduct Target Ther.

[65] Novellasdemunt L, Antas P, Li VS (2015). Targeting Wnt signaling in colorectal cancer. A Review in the Theme: Cell Signaling: Proteins, Pathways and Mechanisms. Am J Physiol Cell Physiol.

[66] Clevers H (2006). Wnt/beta-catenin signaling in development and disease. Cell.

[67] Zhao H, Ming T, Tang S, Ren S, Yang H, Liu M (2022). Wnt signaling in colorectal cancer: pathogenic role and therapeutic target. Mol Cancer.

[68] Ghahhari NM, Babashah S (2015). Interplay between microRNAs and WNT/beta-catenin signalling pathway regulates epithelial-mesenchymal transition in cancer. Eur J Cancer.

[69] Pai P, Rachagani S, Dhawan P, Batra SK (2016). Mucins and Wnt/beta-catenin signaling in gastrointestinal cancers: an unholy nexus. Carcinogenesis.

[70] Medina MA, Ugarte GD, Vargas MF, Avila ME, Necunir D, Elorza AA (2016). Alternative RUNX1 Promoter Regulation by Wnt/beta-Catenin Signaling in Leukemia Cells and Human Hematopoietic Progenitors. J Cell Physiol.

