# Supplementary material for: RUNX1-MUC13 Interaction Activates Wnt/β-Catenin Signaling Implications for Colorectal Cancer Metastasis
Source: Int J Biol Sci. 2024 Sep 16;20(12):4999–5026. doi: 10.7150/ijbs.98396 (PMC11414392; doi:10.7150/ijbs.98396)

Supplementary Table 1. List of the shRNA target sequences.

Gene	Sequences
sh-RUNX1-1	CCTCGAAGACATCGGCAGAAA
sh-RUNX1-2	GAACCAGGTTGCAAGATTTAA
sh-RUNX1-3	GAACCACTCCACTGCCTTTAA
sh-MUC13-1	GACTCGACTGTAAGGACAAAT
sh-MUC13-2	ACCAATGTACAAGCTATTATT
sh-MUC13-3	TTGAGTTAAGTGACCTAATTC
sh-NC	CCTAAGGTTAAGTCGCCCTCG

Supplementary Material Legend

Figure S1. The online database data of RUNX1 were retrieved and statistically analyzed.

(A) Based on the GEPIA database, differences in the expression levels of RUNX1 in various digestive system tumors. **(B, C)** Protein expression level of RUNX1 in CRC was detected using CPTAC (B) and HPA (C) databases. The bar graph (C) shows the results of quantitative analyses. **(D)** Kaplan-Meier survival analysis of COAD patients' disease-free survival (DFS) based on RUNX1 expression in GEPIA. Survival curves were tested by log-rank test. Error bars represent mean ± SEM (n = 3). ****P* < 0.001.

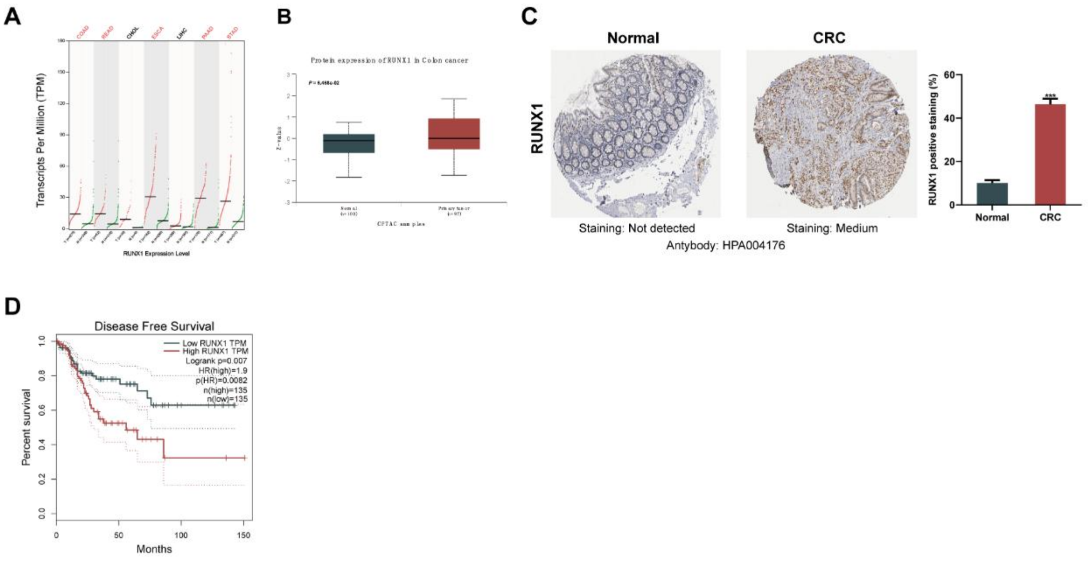

Figure S2. RUNX1 is upregulated in HCC cells.

(A, B) The relative expression levels of RUNX1 measured by qPCR (A) and western blot (B) in five HCC cell lines and a human normal liver cell line, LO-2. ns, not significant; **P* < 0.05; ***P* < 0.01; ****P* < 0.001.

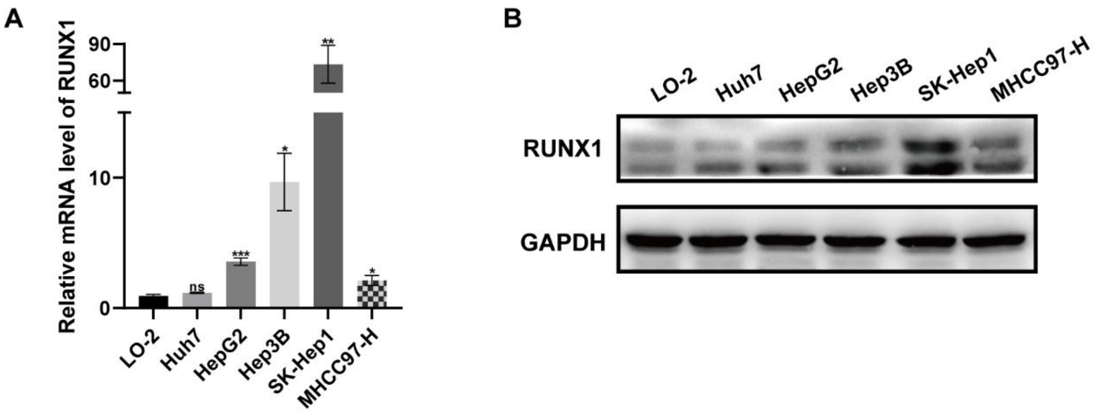

Figure S3. RUNX1 expression in F0 and F3 cells was determined by qPCR (A) and western blotting (B).

Tumor-derived primary cells (F3) derived from three different metastases were used (B). Error bars represent mean ± SEM (n = 3). ***P < 0.001.

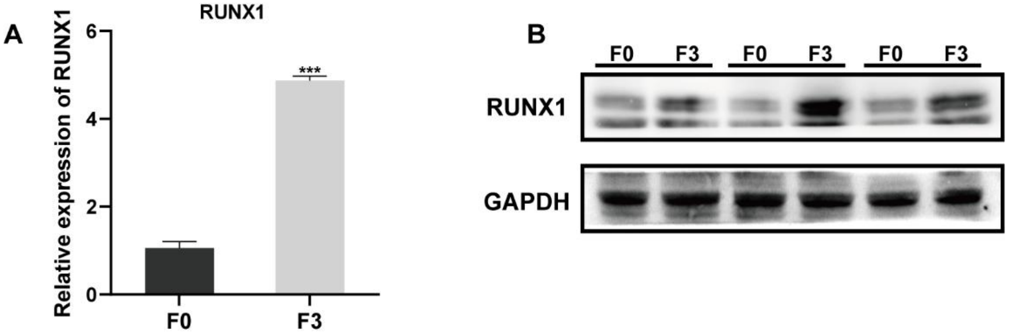

Figure S4. Investigating RUNX1-MUC13 regulatory interactions.

(A) Motifs exhibiting statistically significant differential binding to RUNX1. **(B)** Interaction network diagrams delineate the connections between motifs and transcription factors (TFs), where purple represents the TFs or TF families, and red indicates the target genes potentially regulated by TFs through motifs. **(C)** A heatmap from RNA-Seq analysis presents MUC13 with pronounced upregulation among key differentially expressed genes. **(D)** qPCR validation of the top 13 upregulated genes in RNA-Seq, with MUC13 highlighted in a red box. **P < 0.01, ***P < 0.001, ****P < 0.0001.

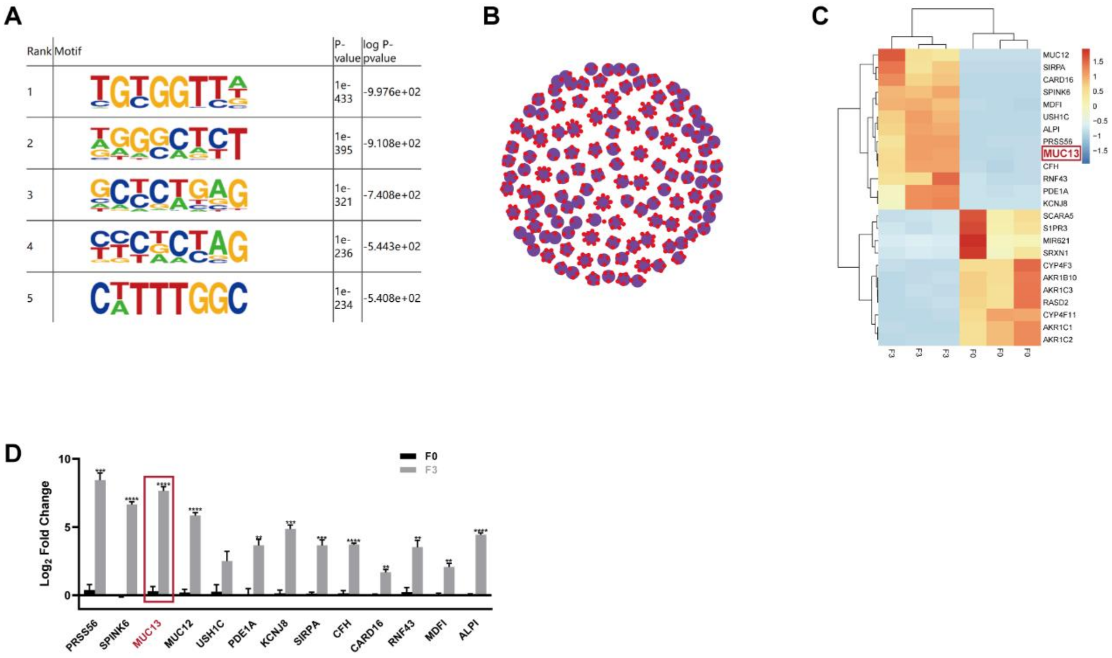

Figure S5. MUC13 is upregulated in HCC cells.

(A, B) The relative expression levels of MUC13 measured by qPCR (A) and western blot (B) in five HCC cell lines and a human normal liver cell line, LO-2. ns, not significant; *$P < 0.05$; **$P < 0.01$.

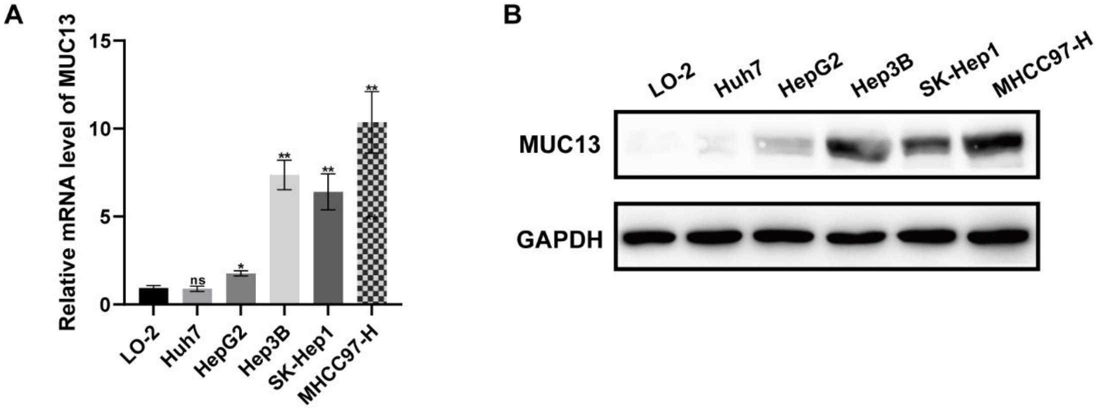

Figure S6. Impact of RUNX1 and MUC13 crosstalk on colorectal cancer cell growth.

(A) IHC staining assessment of RUNX1 and MUC13 protein levels in liver metastatic lesions of mice and the combined impact of these proteins on colorectal cancer cell proliferation via Ki67 staining. Scale bar: 100 μm. **(B)** Correlation analysis between MUC13 and cell proliferation markers based on Spearman correlation analysis from the GEPIA database. R, Spearman coefficient.

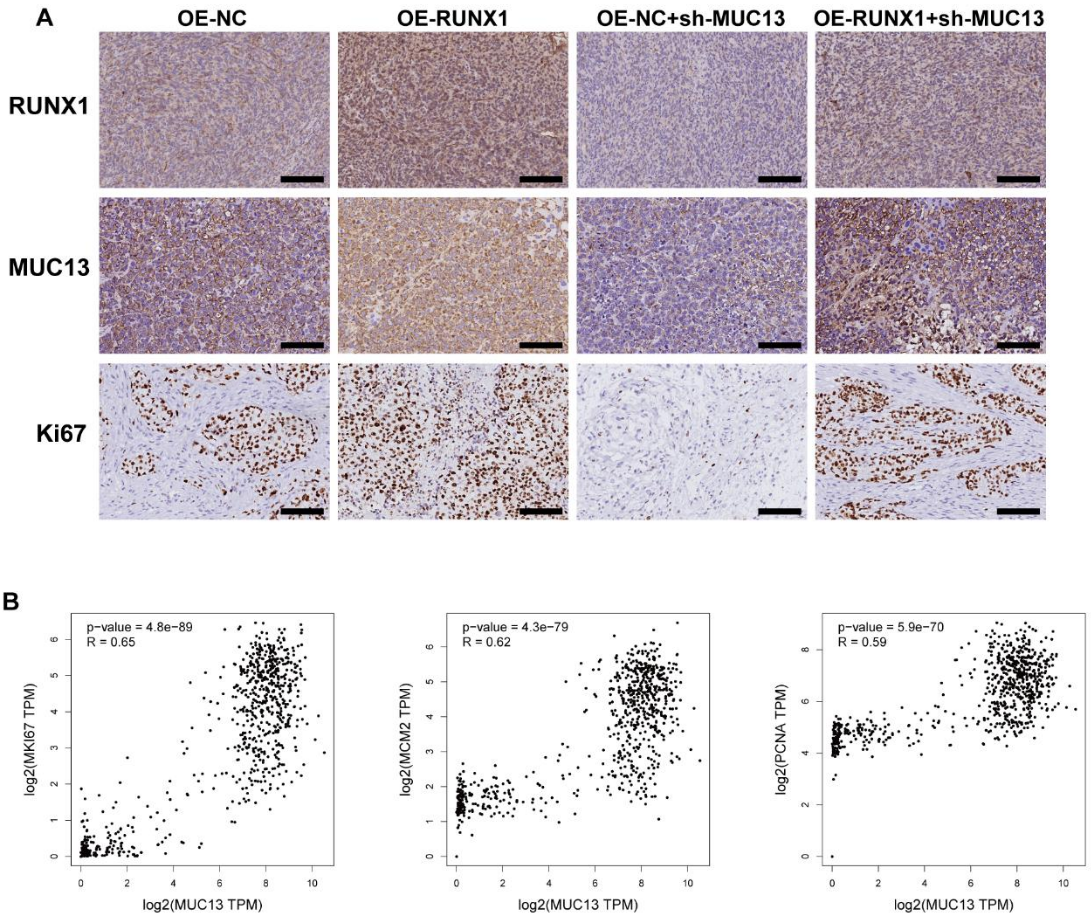

Supplement: Supplementary file 1 — Supplementary figures and tables. [file ijbsv20p4999s1.pdf]
